# GM-CSF augmented the photothermal immunotherapeutic outcome of self-driving gold nanoparticles against a mouse CT-26 colon tumor model

**DOI:** 10.1186/s40824-023-00430-6

**Published:** 2023-10-23

**Authors:** Jie Dai, Jianmei Li, Yuqin Zhang, Qian Wen, Yun Lu, Yu Fan, Fancai Zeng, Zhiyong Qian, Yan Zhang, Shaozhi Fu

**Affiliations:** 1 Department of Oncology, the Affiliated Hospital of Southwest Medical University, Luzhou, 646000 Sichuan P.R. China; 2 Laboratory of Biochemistry and Molecular Biology, School of Basic Medical Sciences, Southwest Medical University, Luzhou, 646000 Sichuan P.R. China; 3 State Key Laboratory of Biotherapy and Cancer Center, West China Hospital, Collaborative Innovation Center for Biotherapy, Sichuan University, Chengdu, 610041 Sichuan P.R. China; 4 Department of Oncology, the Affiliated Traditional Chinese Medicine Hospital of Southwest Medical University, Luzhou, 646000 Sichuan P.R. China; 5 Nuclear Medicine and Molecular Imaging Key Laboratory of Sichuan Province, Luzhou, 646000 Sichuan P.R. China

**Keywords:** Gold nanoparticels, GM-CSF, Anaerobic bacteria, Photothermal therapy, Immunotherapy, Colorectal cancer

## Abstract

**Background:**

Hypoxia is a frequent characteristic observed in solid tumors and is strongly associated with tumor metastasis, angiogenesis, and drug resistance. While the vasculature of hypoxic tumor tissues poses obstacles to the efficient administration of conventional drugs, it may prove advantageous in sustaining hyperthermia. Photothermal therapy (PTT) offers a promising treatment strategy that utilizes the activation of photosensitizers to produce heat, thus facilitating the selective ablation of tumor tissues.

**Method:**

To enhance the accumulation of photothermal agents in tumor tissue and improve the effectiveness of PTT, we developed a self-propelled hybrid called Bif@PAu-NPs. This hybrid consists of polydopamine (PDA)-coated gold nanoparticles (Au-NPs) loaded onto the anaerobic *Bifidobacterium infantis* (Bif).

**Results:**

The Bif@PAu-NPs actively aggregated at the tumor site because the ability of Bif can target hypoxic regions, and PAu-NPs achieved precise PTT due to their high photothermal conversion efficiency (η = 67.8%). The tumor tissues were ablated by PTT, resulting in the release of antigens through immunogenic cell death (ICD), which stimulates an immune response. The inclusion of GM-CSF enhanced the immune response by recruiting dendritic cells and initiating long-term anti-tumor immunity.

**Conclusion:**

The Bif@PAu-NPs hybrid effectively suppressed the growth of both primary tumors and re-challenged tumors. The utilization Bif@PAu-NPs in conjunction with GM-SCF exhibits great potential as a photothermal-immunotherapeutic strategy for precisely treating solid tumors.

**Graphical Abstract:**

In this study, the bacterial Bif@PAu-NPs biohybrid is exploited the self-driving ability of anaerobic Bifidobacterium infantis to deliver polydopamine-modified gold nanoparticles to hypoxic region of tumor. Under irradiation with 808 nm NIR laser, the hybrid exerts precise photothermal therapy to stimulate the immune response, which is further enhanced by GM-CSF, leading to recruitment of dendritic cells and initiation of a long-term anti-tumor immunity remember to prevent tumor recurrence.

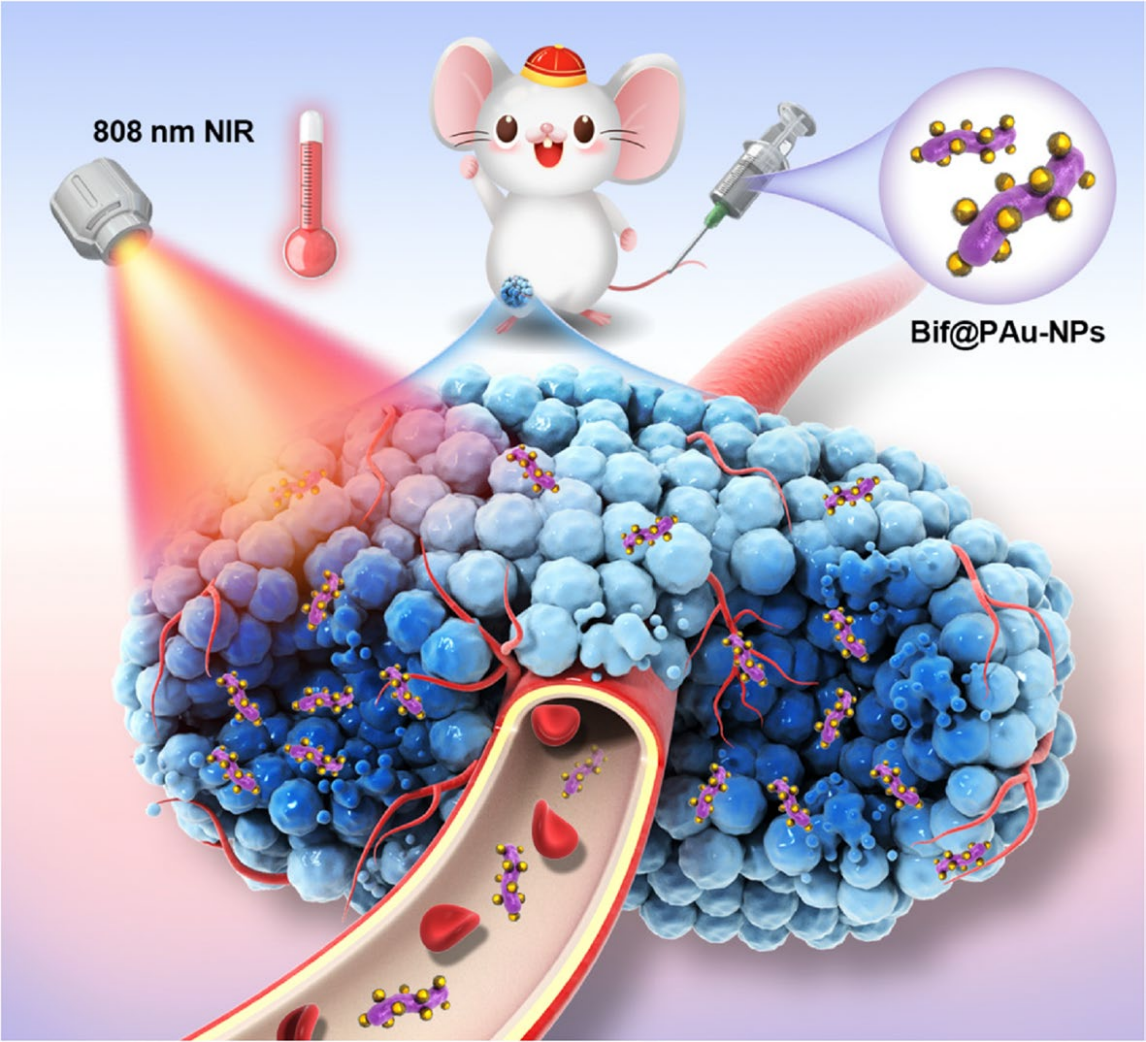

**Supplementary Information:**

The online version contains supplementary material available at 10.1186/s40824-023-00430-6.

## Introduction

Colorectal cancer (CRC) is a prevalent cancer of the gastrointestinal system, and is the second leading cause of cancer-related deaths worldwide [1]. Currently, CRC is treated with surgery, chemotherapy, and radiotherapy, etc [2, 3]. Although these treatments have some therapeutic effects, the clinical cure rate and patient survival are still low due to the limitations of single therapies [4, 5], as well as the high rates of metastasis and recurrence [6]. In recent years, the development of new nanomaterials with diverse physical properties has opened up new treatment opportunities for CRC [7–9]. For example, gold nanoparticles (Au-NPs) have been widely used in photothermal therapy (PTT) due to their excellent biocompatibility and photothermal efficiency [10]. PTT is a local and minimally invasive treatment method, that offers advantages such as spatial and temporal control, targeted therapy, and fewer side effects on normal tissues compared to traditional cancer treatments [6, 11]. PTT works by using a photosensitizer to absorb near-infrared (NIR) light, which is then converted into heat, leading to local hyperthermia and immunogenic cell death (ICD) in the tumor tissue. The damaged tumor cells release damage-associated molecular patterns (DAMP) such as high mobility histone B1 (HMGB1), calreticulin (CRT) and adenosine triphosphate (ATP) [12], which enhance their recognition and phagocytosis by macrophages and dendritic cells (DCs). The mature and activated DCs then present the tumor antigens to T lymphocytes, triggering an adaptive anti-tumor immune response [13, 14]. Despite the widespread use of Au-NPs in targeted PTT [15], they can be toxic at therapeutic concentrations [16]. Additionally, using low concentrations of Au-NPs may require more intense NIR light to maintain hyperthermia, resulting in off-target damage to surrounding tissues. Coating the particles with polydopamine (PDA), which is formed through the oxidation of dopamine under alkaline conditions can attenuated the toxicity of Au-NPs [17]. Dopamine is derived from adhesion proteins secreted by invertebrate mussels [18]. PDA has several advantages compared to other modifiers, including serving as a drug delivery carrier and a photothermal agent [19–21], as well as having good biocompatibility and biodegradability due to its chemical and structural similarity to melanin [22, 23]. PDA also exhibits strong adhesion properties because of the presence of catechol amino acids like 3,4-dihydroxy-L-phenylalanine [24, 25]. Although PDA modification can improve the photosensitivity, selectivity, biocompatibility and activity of Au-NPs, the effectiveness of PTT is still limited by the poor accumulation of photothermal agents in the tumor. Therefore, it is crucial to enhance the targeted delivery of photothermal agents in order to improve their accumulation at tumor sites.

Hypoxia is one of the typical hallmarks of the tumor microenvironment (TME) [26, 27]. Studies have shown that anaerobic bacteria can specifically target and colonize the hypoxic areas of solid tumors [28–31]. We have previously confirmed that drug-loaded nanoparticles (NPs) can bind to *Bifidobacterium infantis* (*B. infantis*, Bif) to form a biohybrid for targeted tumor treatment [32, 33]. *B. infantis* (Bif), which is a harmless anaerobic probiotic with no apparent toxic side effects and excellent targeting abilities [34, 35], is thus considered an ideal therapy vector for treating different types of solid tumors [36]. However, due to the limited penetration depth of NIR light, NPs-mediated PTT is only effective against primary tumors, and tumors outside the range of laser irradiation are not completely eliminated. Therefore, PTT alone is insufficient to eradicate distal metastases [37, 38]. While immunotherapy shows potential advantages in treating metastatic and recurrent tumors [39], the weak immunogenicity of tumor antigens and low expression of molecules related to T-cell activation hinder the induction of an effective anti-tumor immune response [40]. PTT-induced tumor cell death can release tumor antigens and enhance immunotherapy [41–43], while immunotherapy can also enhance ICD to further improve systemic anti-tumor immunity. Granulocyte-macrophage colony-stimulating factor (GM-CSF), which is produced by macrophages and activated T cells, plays a crucial role in the anti-tumor response [44]. It promotes the differentiation, proliferation, and recruitment of dendritic cells (DCs) [45]; enhances antigen presentation and co-stimulatory molecule expression; and stimulates pro-inflammatory cytokine production [46]. Therefore, GM-CSF has been used to enhance DCs-mediated anti-tumor immunity [47].

**Scheme 1:**
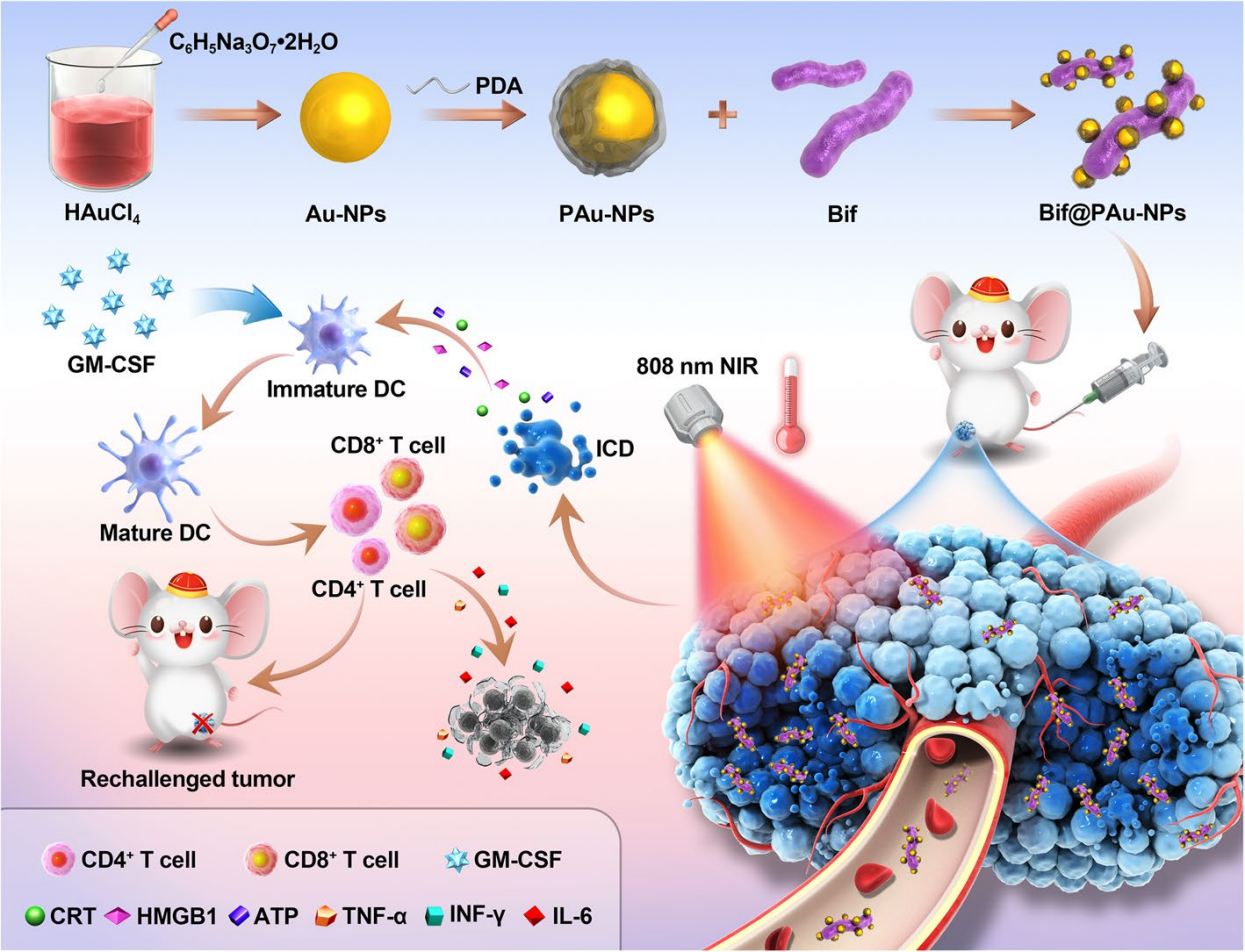
Schematic illustration of the synthesis and antitumor performance of Bif@PAu-NPs

In our study, we designed bacterial hybrid Bif@PAu-NPs to transport Au-NPs for targeted photothermal immunotherapy against CRC (Scheme 1). The Bif@PAu-NPs, when intravenously injected, can actively accumulate in tumor tissues due to the specific self-driving ability of Bif towards hypoxic regions. Furthermore, the photothermal effect caused by Au-NPs and PDA in response to 808 nm NIR light irradiation triggers ICD, as described, and the resulting immune response is further enhanced by GM-CSF. The use of Bif@PAu-NPs in combination with GM-CSF induces long-term immune memory, thereby preventing tumor recurrence and metastases.

## Materials and methods

### Reagents and biological materials

Chloroauric acid (HAuCl_4_) was purchased from Xiangbo Biotechnology Co. (Guangzhou, China). Dopamine hydrochloride, trisodium citrate, thiazolyl blue bromide (MTT), DMSO, and the Calcein/PI cell activity and cytotoxicity assay kits were provided by Beyotime Biotechnology (Shanghai, China). Fetal bovine serum (FBS), DMEM, mouse TNF-α, INF-γ, IL-6, CRT, HMGB1 and ATP ELISA kits were purchased from Xinkesheng Biotechnology Co. Ltd. (Luzhou, China). PE anti-mouse CD11c antibody, APC anti-mouse CD86 antibody, and Annexin V-FITC/PI apoptosis assay kit were from Becton, Dickinson Co. Ltd.

The CT-26, 4T1, A549 and AML-12 cell lines were purchased from the Cell Bank of the Chinese Academy of Sciences (Shanghai, China). *B. infantis* (GIMI.207) was purchased from Guangdong Microbial Culture Collection Center (Guangzhou, China). Male BALB/c mice weighing 16–18 g (6 weeks old) were purchased from Tengxin Bill Laboratory Animal Sales Co. Ltd. (Chongqing, China). All animal experiments were approved by the ethical and scientific committee of the Animal Care and Treatment Committee of Southwest Medical University (SWMU20220026).

### Preparation and characterization of Au-NPs, PAu-NPs, and Bif@PAu-NPs

The sodium citrate reduction method was used to prepare Au-NPs [48]. Briefly, 100 mL of 0.01% chloroauric acid was brought to boiling and then mixed with 4 mL of 1% trisodium citrate dihydrate. The mixture was heated until it turned a burgundy color, and then cooled to room temperature. After centrifugation at 10,000 rpm for 10 min, the resulting Au-NPs were freeze-dried in a vacuum and stored at 4 °C. The morphology of the Au-NPs was observed using transmission electron microscopy (TEM, FEI Tecnai G2 F30 USA). Particle size and zeta potential were measured using dynamic light scattering (DLS, NanoBrook90 plus Zeta, Brookhaven, NY) at 25 °C.

PDA coated Au-NPs (PAu-NPs) were prepared through oxidative self-polymerization [49]. To elaborate, 50 mg of Au-NPs and 20 mg of dopamine hydrochloride were dissolved in 50 mL of Tris-HCl buffer (10 mM, pH = 8.5), and stirred in the dark for 6 h. The solution was then centrifuged at 15,000 rpm for 10 min to obtain PAu-NPs, and their morphology, particle size and zeta potential were characterized.

To create Bif@PAu-NPs hybrids, a 1 mg/mL suspension of PAu-NPs was incubated with Bif (2 × 10^7^ CFU/mL) at 37 °C for 4 h. The mixture was then centrifuged at 2500 rpm for 5 min, and the precipitate was washed twice with PBS (pH = 7.4) to obtain Bif@PAu-NPs hybrids. Hybrids labeled with Nile Red (NR) (Bif@PDA-NR-NPs) were prepared in a similar manner. The morphology and energy spectra of the Bif@PAu-NPs were observed using a scanning electron microscope (SEM, SU8020 Hitachi, Japan), and their elemental distribution was evaluated using high-angle annular dark field scanning transmission electron microscopy (HAADF-STEM, FEI Tecnai G2 F30 USA). Physical phase was assessed using X-ray diffraction (BRUCKER D8 ADVANCE Germany) and thermogravimetric analysis (TGA, NETZSCH STA 449 F5/F3 Jupiter Germany). UV absorption spectra of Au-NPs, PAu-NPs, Bif, Bif@PAu-NPs were also analyzed. To evaluate the impact of PAu-NPs on bacterial growth, freshly prepared Bif@PAu-NPs and Bif were incubated for 48 h in an anaerobic environment, and the number of viable bacterial cells was counted.

The in vitro stability of the Bif@PAu-NPs hybrids was investigated by incubating them in an acidic solution (pH = 6.5) with a high concentration of reductive glutathione (GSH, 10 mM) for 4 h to simulate the tumor microenvironment. The incubation in a GSH-free solution at a pH of 7.4 was used as a control. After centrifugation, the UV absorbance of the supernatants was measured using a UV-Vis spectrophotometer (UV-5800PC, Shanghai Metash Instruments Co. Ltd., Shanghai, China).

### Photothermal properties of Au-NPs

#### Photothermal effect

To determine the optimal power and concentration of the nanoparticles for PTT, different concentrations (25, 50, 100 and 200 µg/mL) of a 1 mL aqueous solution of PAu-NPs were exposed to an 808 nm NIR laser (2 W/cm^2^) for 5 min. The temperature of the solution was measured every 30 s using an infrared thermographer (Fluke, Ti75+, USA). Additionally, a 200 µg/mL solution of PAu-NPs was exposed to the NIR laser at various power densities (0.5, 1, 1.5 and 2 W/cm^2^), and the temperatures of the solutions were recorded as described.

#### Photothermal stability

The aqueous solution of PAu-NPs (200 µg/mL) was continuously exposed to an 808 nm laser at 2 W/cm^2^ for 5 min and subsequently cooled to room temperature. The temperature was recorded every 30 s, and this cycle was repeated three times.

#### Photothermal conversion efficiency

The PAu-NPs solution (200 µg/mL) was irradiated with an NIR laser (808 nm, 2 W/cm^2^, 5 min) and the temperature was recorded. The photothermal conversion efficiency (η) was calculated using the following Eq. (1), as previously described [50]:

1 $$\eta =\frac{\text{h}\text{S}\left({T}_{\text{m}\text{a}\text{x}}-{T}_{ssur}\right)-{Q}_{dis}}{\text{I}\left(1-{10}^{-{A}_{808}}\right)}$$

Where T_max_ is the maximum equilibrium temperature, T_surr_ is the surrounding ambient temperature, Q_dis_ is the heat loss of light absorbed by the vessel, I (W/cm^2^) represents the incident laser power, A_808_ is the absorbance of the sample at 808 nm, h (W/cm^2^·K) represents the heat transfer coefficient, and S (cm^2^) represents the surface area of the vessel. The value of hs was calculated by Eq. (2) as follows:

2 $${\tau }_{s}=\frac{{\text{m}}_{D}{c}_{D}}{hs}$$

Where τs is the time constant of the sample system, and m_D_ and c_D_ are respectively the mass (1 g) and heat capacity (4.2 J/g·°C) of the solvent.

### In vitro biological assays

#### Cellular uptake and cytotoxicity

CT-26 cells were seeded in 6-well plates and incubated with normal saline (NS), Nile Red (NR), NR-loaded NPs (NR-NPs), or PDA-NR-NPs for 3 h. After staining with DAPI, the cells were observed under a fluorescence microscope (OLYMPUS, IX73, Japan) to assess the uptake of different particles. CT-26 and AML-12 cells (5 × 10^3^ cells) were incubated with Au-NPs and PAu-NPs for 24 h, and then irradiated with 808 nm NIR light (2 W/cm^2^) for 5 min. After 24 h, 20 µL of MTT solution (5 mg/mL) and 150 µL of DMSO were added to each well, and the optical density at 490 nm was measured using a FLUOstar Omega microplate reader.

#### Calcein-AM/PI assay

CT-26, 4T1 and A549 cells were incubated with 0.5 mL of NS, Au-NPs or PAu-NPs (200 µg/mL) for 4 h. After irradiation with 808 nm laser (2 W/cm^2^) for 5 min, the cells were further incubated further for 2 h. The suitably treated cells were gently washed and stained with Calcein-AM (1 µg/mL) and pyridinium iodide (PI) (1 µg/mL) for 30 min. The viable cells emitting green fluorescence (λex = 490 nm, λem = 515 nm) and dead cells with red fluorescence (λex = 535 nm, λem = 617 nm) were counted using a fluorescence microscope (OLYMPUS, IX73, Japan).

#### Apoptosis assay

CT-26 cells and AML-12 cells were seeded at a density of 5 × 10^4^ cells/well and incubated with 0.5 mL of NS, Au-NPs or PAu-NPs at a concentration of 200 µg/mL for 4 h. Some wells were exposed to an 808 nm laser at an intensity of 2 W/cm^2^ for 5 min. After incubation, the cells were stained with 5 µL of Annexin V-FITC and 5 µL of propidium iodide (PI) in the dark for 15 min. Apoptosis was measured using flow cytometry (BD FACSVerse, Piscataway, NJ).

#### Hemolysis assay

To evaluate hemolysis, 1 mL of erythrocyte suspension (2%, v/v) was mixed with 1 mL of PAu-NPs, Bif or Bif@PAu-NPs. Double distilled water and saline were used as positive and negative controls, respectively. All samples were incubated at 37 °C for 4 h and then centrifuged at 3000 rpm for 5 min. The optical density (OD) of the supernatant was measured at 540 nm using a UV-Vis spectrophotometer (UV-5800PC, Shanghai Metash Instruments Co. Ltd., Shanghai, China). The hemolysis rate was calculated according to the following Eq. (3)

3 $$\text{Hemolysis Rate }\left({\%}\right)=\frac{OD\text{ value of experimental group }-OD\text{ value of saline group }}{\text{ OD value of positive control group }-OD\text{ value of saline group }}\times 100{\%}$$

#### Evaluation of the hypoxia tropism of Bif

To verify the targeting ability of Bif to hypoxic environment, bacterial migration was assessed using transwell chambers. A suspension of Bif (200 µL, 5 × 10^7^ CFU/mL) was seeded into the upper chamber of the transwell insert, while the lower chamber was filled with 400 µL mixture of glucose (0.4 mg/mL), glucose oxidase (0.5 kU) and catalase (0.5 kU). Glucose oxidation depletes oxygen and produces hydrogen peroxide, which is then quenched by catalase, creating an artificial hypoxic environment in vitro. The control wells maintained a normoxic condition in the bottom chamber. After 2 h of incubation, the number of bacteria that migrated to the bottom chamber was counted.

### In vivo evaluation of anti-tumor efficacy

CT-26 cells (0.2 mL, 5 × 10^6^) were subcutaneously implanted into the right leg of male Balb/c mice. When tumor volume reached 50–80 mm^3^, the mice were randomly divided into six groups as follows (*n* = 6 per group): NS, Bif + NIR + GM-CSF, PAu-NPs + NIR + GM-CSF, Bif@PAu-NPs + GM-CSF, Bif@PAu-NPs + NIR and Bif@PAu-NPs + NIR + GM-CSF groups (Bif: 2 × 10^7^ CFU/mL, PAu-NPs: 2.5 mg/kg, GM-CSF:1.5 mg/kg). The mice were intravenously injected with 100 µL of the drugs and irradiated with NIR laser (808 nm, 2 W/cm^2^, 5 min) 24 h after injection. The temperature was monitored and controlled at approximately 45 °C using an infrared thermography instrument (Fluke, Ti75+, USA). GM-CSF was intravenously injected 24 h after laser irradiation. Tumor volumes were measured during treatment. At the end of the treatment, the mice were euthanized and the tumor tissues were imaged and processed for TUNEL staining, hematoxylin-eosin (HE) staining and immunohistochemistry (CD4 and CD8). Serum samples were collected for ELISA (CRT and HMGB1).

#### In vivo biodistribution of Bif

The CT-26 tumor-bearing mice were intravenously injected with Bif@PAu-NPs (0.1 mL) once the tumor volume reached 50 cm^3^. The mice were euthanized on days 1, 4, 7, and 14, and the tumors along with the major organs (heart, liver, spleen, lung, and kidney) were harvested. The tissue samples were homogenized in sterile water with 0.1% Triton X-100, spread onto LB agar plates, and incubated anaerobically at 37 °C for 48 h. The number of Bif@PAu-NPs was then counted.

Tumor-bearing mice were intravenously injected with Bif@PAu-NPs or Bif (100 µL, 2 × 10^7^ CFU/mL), and euthanized 24 h later. The tumor tissues were harvested, embedded in paraffin, and sectioned. The sections were incubated overnight with primary antibodies anti-Bif (1:25) and anti-HIF-1α (1:100) primary antibodies at 4 °C. Then secondary antibodies (goat anti-mouse FITC-conjugated (1:300) and goat anti-rabbit Cy3-conjugated (1:400)) were applied for 50 min at room temperature in the dark. DAPI solution was used for counterstaining for 10 min. The slides were observed under a fluorescent microscope and the distribution of Bif was statistically analyzed.

#### In vivotargeting ability of Bif@PAu-NPs

To synthesize Bif@PAu-NPs labeled with indocyanine green(ICG), a Bif@PAu-NPs solution (5 mL, 1 mg/mL) was incubated with 5 mg of ICG at room temperature in the dark for 24 h. The labeled Bif@PAu-NPs were then centrifuged and washed with saline for further use. CT-26 tumor-bearing mice were randomly divided into 4 groups (*n* = 3) and injected with free ICG (1 mg/kg), Bif@ICG (1 mg/kg, 2 × 10^7^ CFU/mL), PAu-NPs/ICG (1 mg/kg) or Bif@PAu-NPs@ICG (1 mg/kg, 2 × 10^7^ CFU/mL). The mice were imaged at 6, 12, 24 and 48 h after injection using a multimodal small animal live imaging system (ThermoFisher FXPRO USA). After the last time point, the mice were euthanized. Subsequently, the tumors and major organs were harvested for ex vivo imaging.

To determine the biodistribution of Au-NPs, CT-26 tumor-bearing mice were randomly divided into 3 groups (*n* = 5) and injected with 100 µL of PAu-NPs (50 µg/kg), Bif (2 × 10^7^ CFU/mL) or Bif@PAu-NPs (50 µg/kg, 2 × 10^7^ CFU/mL). After 24 h, the mice were euthanized. Subsequently, the tumor tissues and major organs were weighed, and then digested in aqua regia at 80 °C. The gold content in the tissue samples was measured using inductively coupled plasma mass spectrometry (ICP-MS) (Agilent ICPMS7800 USA).

#### Microscopic PET/CT scanning

Whole-body microPET/CT scans (Siemens Germany) were conducted to evaluate the early response of the mice to the different formulations. Three mice were chosen randomly from each group 48 h after the final treatment. After fasting for 6 h, 200–250 µCi of 18 F-FDG was injected via the tail vein. Thirty minutes later, the mice were anesthetized using isoflurane and scanned using parameters of 80 kV, 500 mA, and 1.5 mm slice collimation. The region of interest (ROI) on PET/CT images was manually delineated, and the maximum normalized uptake value (SUVmax) and mean uptake value (SUVmean) were calculated.

#### In vivo anti-tumor immune assay

Freshly isolated tumor tissues were homogenized into single-cell suspensions, which were then stained with anti-CD3 (FITC), anti-CD45 (FITC), anti-CD8 (APC), anti-CD4 (PE), anti-CD11b (Percp), anti-CD11c (PE) and anti-CD86 (APC) antibodies according to the manufacturer’s protocol (BD Pharmingen). The stained cells were analyzed using flow cytometry (BD FACSAria USA). Serum samples were diluted, and the levels of TNF-α, IFN-γ, IL-6 and ATP were measured using specific ELISA kits according to the manufacturer’s instructions.

#### In vivo validation of long-term immunological memory

To evaluate long-term immunological memory, primary tumors were established in the right leg using the method described above. Once the tumor reached a size of 100 mm^3^, the mice were randomly divided into 4 groups and treated with NS, Bif@PAu-NPs, Bif@PAu-NPs + NIR and Bif@PAu-NPs + GM-CSF + NIR. After 60 days of treatment, the mice were reinoculated with 5 × 10^6^ CT-26 cells in the left leg to establish a secondary tumor. Body weight and tumor volume were measured every two days after reinoculation.

#### In vivo biocompatibility of Bif@PAu-NPs

Healthy Kunming mice were injected intravenously with NS and Bif@PAu-NPs (50 mg/kg) three times every 2 days. After 14 days, blood samples were collected to measure blood routine indices and biochemical indicators such as red blood cells (RBC), white blood cells (WBC), platelets (PLT), hemoglobin (HGB), mean red blood cell hemoglobin concentration (MCHC), red blood cell-specific volume (HCT), mean hemoglobin (MCH), mean hematocrit (MCV), neutrophil count (NEU), glutamate transaminase (ALT), aspartate transaminase (AST), urea (UREA), glucose (GLU), albumin (ALB), and total cholesterol (TC).

### Statistical analysis

All data were expressed as the mean ± standard deviation (SD) of three independent experiments. The data were evaluated using the Student’s t-test unless otherwise stated. Survival curves were plotted using the Kaplan-Meier method, and survival times and 95% confidence intervals were compared using the log-rank test. Statistical analysis was performed using GraphPad Prism 9 software. A significance level of *P* < 0.05 was considered statistically significant.

## Results

### Preparation and characterization of different nano-gold formulations

**Fig. 1:**
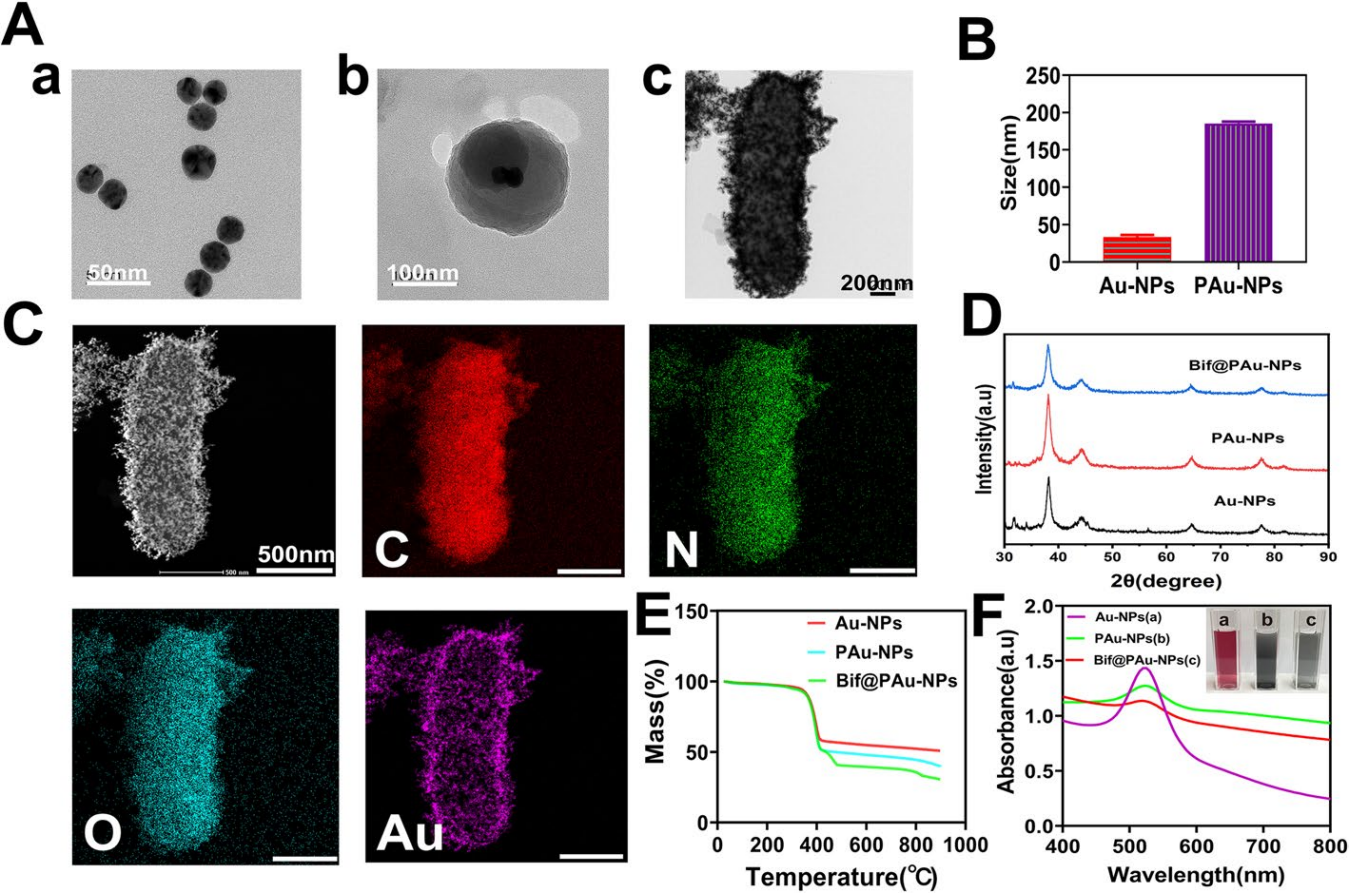
Characterization of Au-NPs, PAu-NPs and Bif@PAu-NPs. **A** TEM images of (a) Au-NPs, (b) PAu-NPs and (c) Bif@PAu-NPs hybrids. **B** Average particle size of Au-NPs and PAu-NPs (*n* = 3). **C** HAADF-STEM images and elemental mapping of Bif@PAu-NPs. **D** XRD patterns of Au-NPs, PAu-NPs and Bif@PAu-NPs. **E** TGA curves of Au-NPs, PAu-NPs and Bif@PAu-NPs. **F** UV-Vis absorption spectra and the color (insert) of Au-NPs (a), PAu-NPs (b) and Bif@PAu-NPs (c) solutions

The monodispersed Au-NPs still maintained their spherical shape even after being coated with PDA (PAu-NPs, Fig. 1Aa-b). Additionally, the PAu-NPs tightly adhered to *B. infantis* (Bif) to form Bif@PAu-NPs (Fig. 1Ac, Figure S1). The diameter of spherical Au-NPs (Fig. 1Aa) increased from 33.41 ± 2.76 nm to 185.50 ± 2.08 nm after PDA coating (Fig. 1B). The zeta potential slightly decreased from − 36.88 ± 0.40 mV to -40.60 ± 2.61 mV) (Figure S2). HAADF-STEM images confirmed that gold was uniformly dispersed on the surface of Bif@PAu-NPs hybrids (Fig. 1C), and STEM-EDS (Figure S3) showed that carbon (C) accounted for 57.49% and gold accounted for 35.72%. Furthermore, X-ray diffraction (XRD) analysis further confirmed the successful preparation of bacterial hybrids by revealing characteristic peaks of gold in Au-NPs, PAu-NPs and Bif@PAu-NPs (Fig. 1D, Figure S4). TGA analysis indicated that gold loading on Bif@PAu-NPs was approximately 40% (Fig. 1E). Finally, the UV-Vis absorption spectra confirmed that the presence of the absorbance peak at 520 nm for Au-NPs in PAu-NPs and Bif@PAu-NPs (Fig. 1F).

The in vitro stability analysis showed that the PDA coating in Bif@PAu-NPs biohybrids can be disrupted in a slightly acidic tumor environment (pH = 6.5) with a high concentration of GSH (10 mM), leading to the release of Au-NPs (Figure S5).

### In vitro photothermal performance of Bif@PAu-NPs

**Fig. 2:**
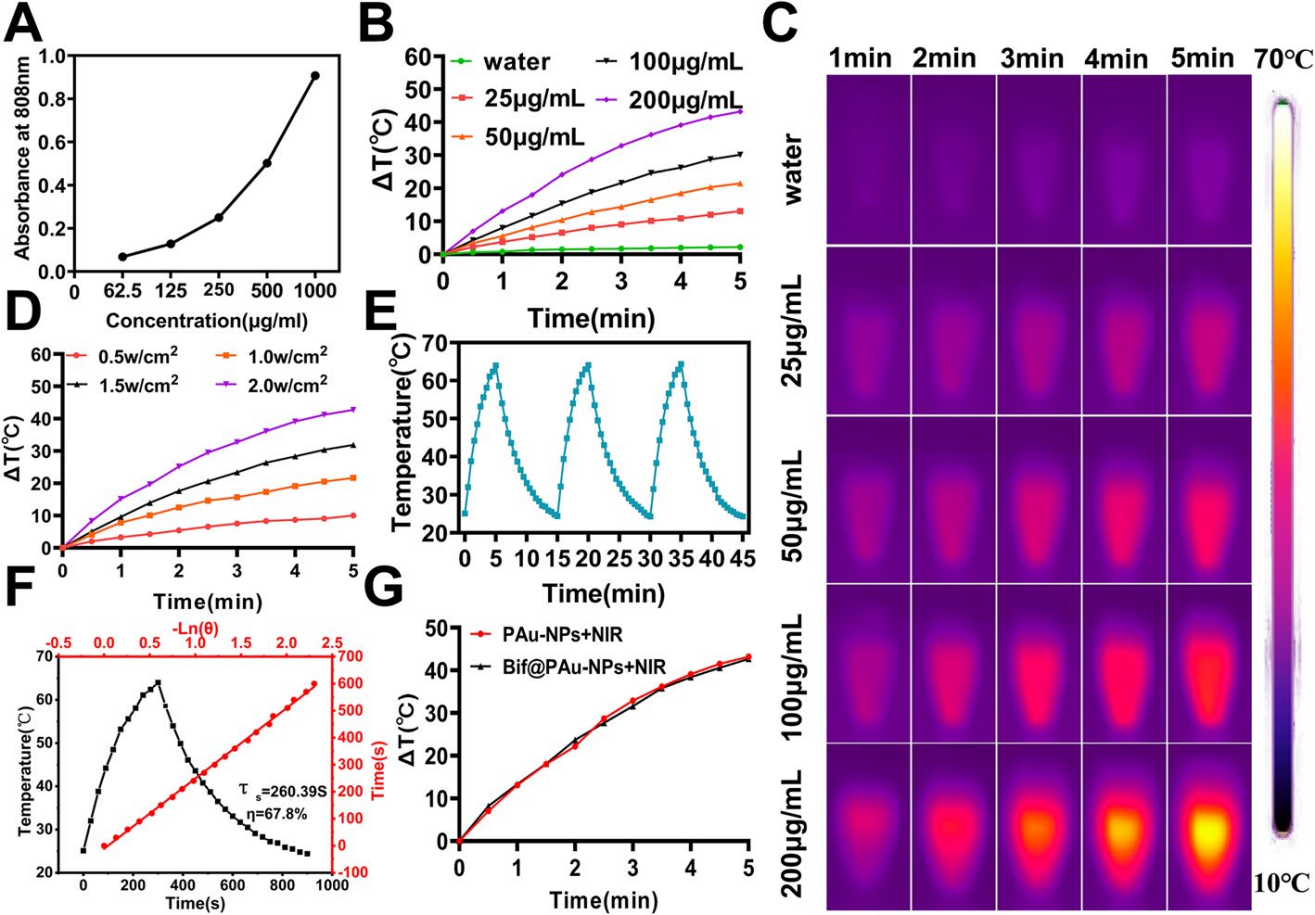
In vitro characterization of photothermal properties. **A** The relative absorbance intensity of PAu-NPs solution with different concentrations at 808 nm NIR irradiation. **B** Heating curves of PAu-NPs at concentrations of 0, 25, 50, 100, 200 µg/mL under 808 nm laser. **C** Infrared thermal images of PAu-NPs solutions under 808 nm laser. **D** Heating curves of PAu-NPs irradiated with different laser powers (0.5, 1, 1.5, 2.0 W/cm^2^). **E** Photothermal stability of PAu-NPs (200 µg/ml, 2.0 W/cm^2^) in three heating-cooling cycles. **F** Heating and cooling curve of PAu-NPs solution under 808 nm laser irradiation (2.0 W/cm^2^). **G** Heating curves of PAu-NPs and Bif@PAu-NPs solutions under 808 nm laser irradiation (2.0 W/cm^2^)

The absorption peak of PAu-NPs at 520 nm increased and the color of the solutions became more intense as the concentration increased (Figure S6-S7). Additionally, the fluorescence intensity at 808 nm also increased in a concentration-dependent manner (Fig. 2A), indicating that PAu-NPs are effective photothermal agents. Figure 2B shows that the temperature of the PAu-NPs solutions significantly increased as the concentration increased from 25 µg/mL to 200 µg/mL. The temperature increase of the PAu-NPs depended on both the concentration and radiation time (Fig. 2C). Moreover, the maximum temperature increase was observed with the increase in laser power (Fig. 2D, Figure S8). Based on the results, it was found that irradiating 200 µg/mL PAu-NPs with an 808 nm NIR laser at 2 W/cm^2^ for 5 min showed the optimal photothermal effect. Furthermore, no energy loss occurred during three heating-cooling cycles of the PAu-NPs, indicating good photothermal stability (Fig. 2E). The photothermal conversion efficiency (η) of the PAu-NPs was found to be 67.8% (Fig. 2F). In addition, there was no significant difference in the temperature difference (ΔT) between PAu-NPs and Bif@PAu-NPs (Fig. 2G), suggesting that the binding of PAu-NPs to the bacterial surface had minimal impact on its photothermal performance. Overall, the Bif@PAu-NPs are suitable photosensitizers for PTT.

### Bif@PAu-NPs achieved PTT in vitro

**Fig. 3:**
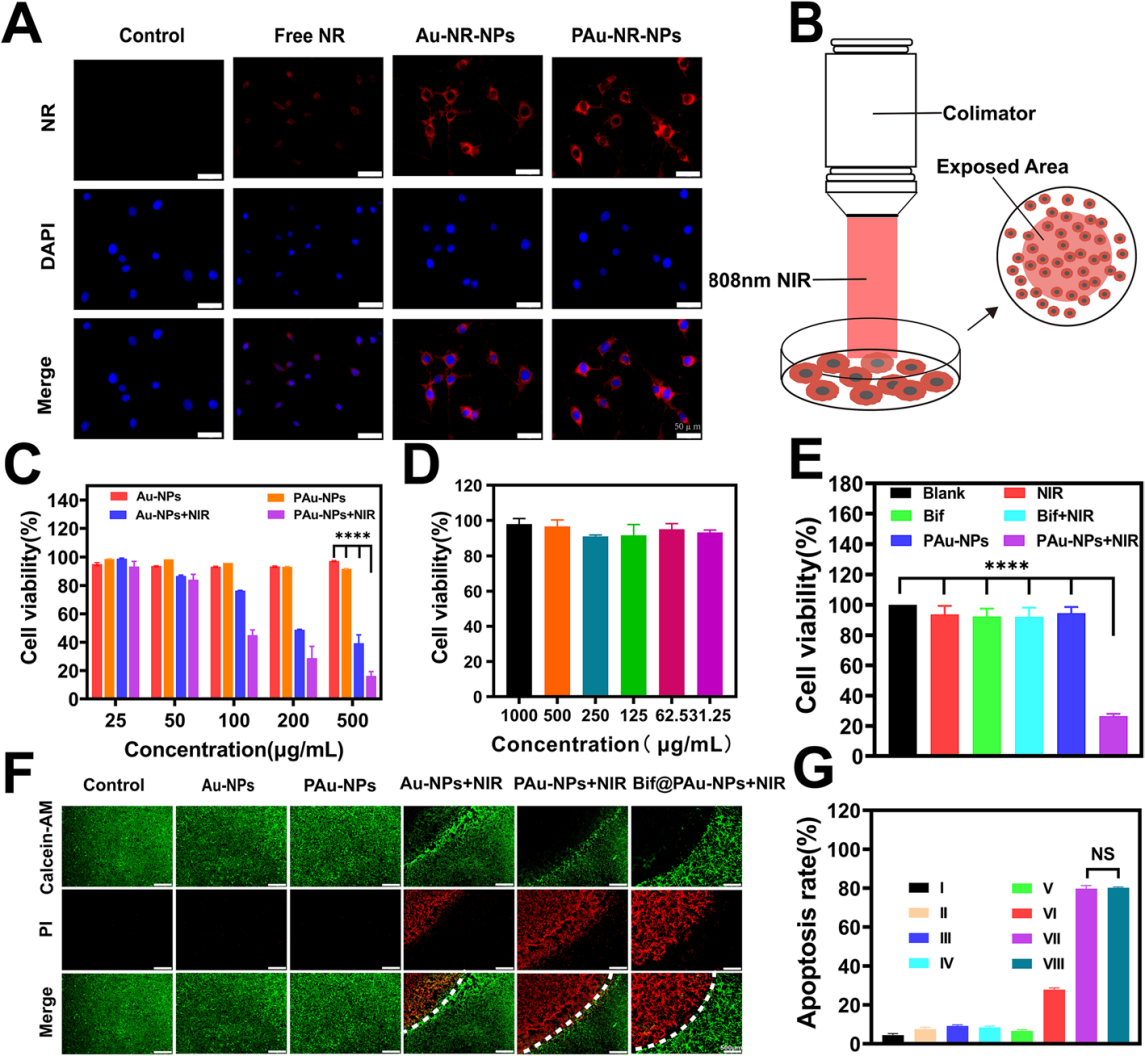
In vitro cellular experiments. Cellular uptake of in vitro photothermal therapy (**A**) Cell uptake of NR-labeled nanoparticles (scale bar 50 μm). **B** Schematic diagram of in vitro PTT. **C** Cell viability of CT-26 cells. (*n* = 3). **D** cell viability of AML-12 cells incubated with Bif@PAu-NPs for 24 h (*n* = 3). **E** Cell viability of CT-26 cells with different treatments. **F** Live/dead staining of CT-26 cells with Calcein AM (green, live cells), PI (red, dead cells). The white dashed line represents the boundary line of laser irradiation. Scale bar = 500 μm. **G** Apoptosis rate of each group. I: Control; II: Au-NPs; III: PAu-NPs; IV: Bif@PAu-NPs; V: NIR; VI: Au-NPs + NIR; VII: PAu-NPs + NIR; VIII: Bif@PAu-NPs + NIR. *P < 0.05, **P < 0.01, ***P < 0.001, ****P < 0.0001

As shown in Fig. 3A, the NR-labeled Au-NPs (Au-NR-NPs and PAu-NR-NPs) were readily taken up by CT-26 cells. Additionally, the PAu-NPs, in combination with NIR irradiation, significantly reduced the viability of tumor cells, with approximately 90% of cells remaining viable in the absence of laser irradiation (Fig. 3B-C). However, even at high concentrations (up to 1000 µg/mL), Bif@PAu-NPs showed minimal cytotoxicity in the normal AML-12 hepatocyte cell line (Fig. 3D). When compared to the other formulations, the apoptosis rate induced by Bif@PAu-NPs in AML-12 cells (9.75%) was not significantly different (P>0.05, Figure S9). As shown in Fig. 3E, neither NIR laser irradiation nor Bif treatment alone significantly affected cell growth, suggesting that the cytotoxicity of PAu-NPs was dependent on photothermal conversion. Moreover, staining with Calcein AM (green, representing live cells) and PI (red, representing dead cells) indicated substantial cell death in the PAu-NPs + NIR and Bif@PAu-NPs + NIR groups compared to the Au-NPs + NIR group. Conversely, no dead cells were observed in the control, Au-NPs, and PAu-NPs groups (Fig. 3F). These results also demonstrated that the formulation of the Bif@PAu-NPs hybrid did not compromise the photothermal efficiency of Au-NPs. The same phenomenon was observed in A549 cells (Figure S10) and 4T1 cells (Figure S11). The apoptosis rates of CT-26 cells in the PAu-NPs + NIR group and Bif@PAu-NPs + NIR group were 79.73% and 80.25% respectively, significantly higher than those of the other groups (Fig. 3G, Figure S12).

### Bif@PAu-NPs specifically targeted the hypoxic tumor regions

**Fig. 4:**
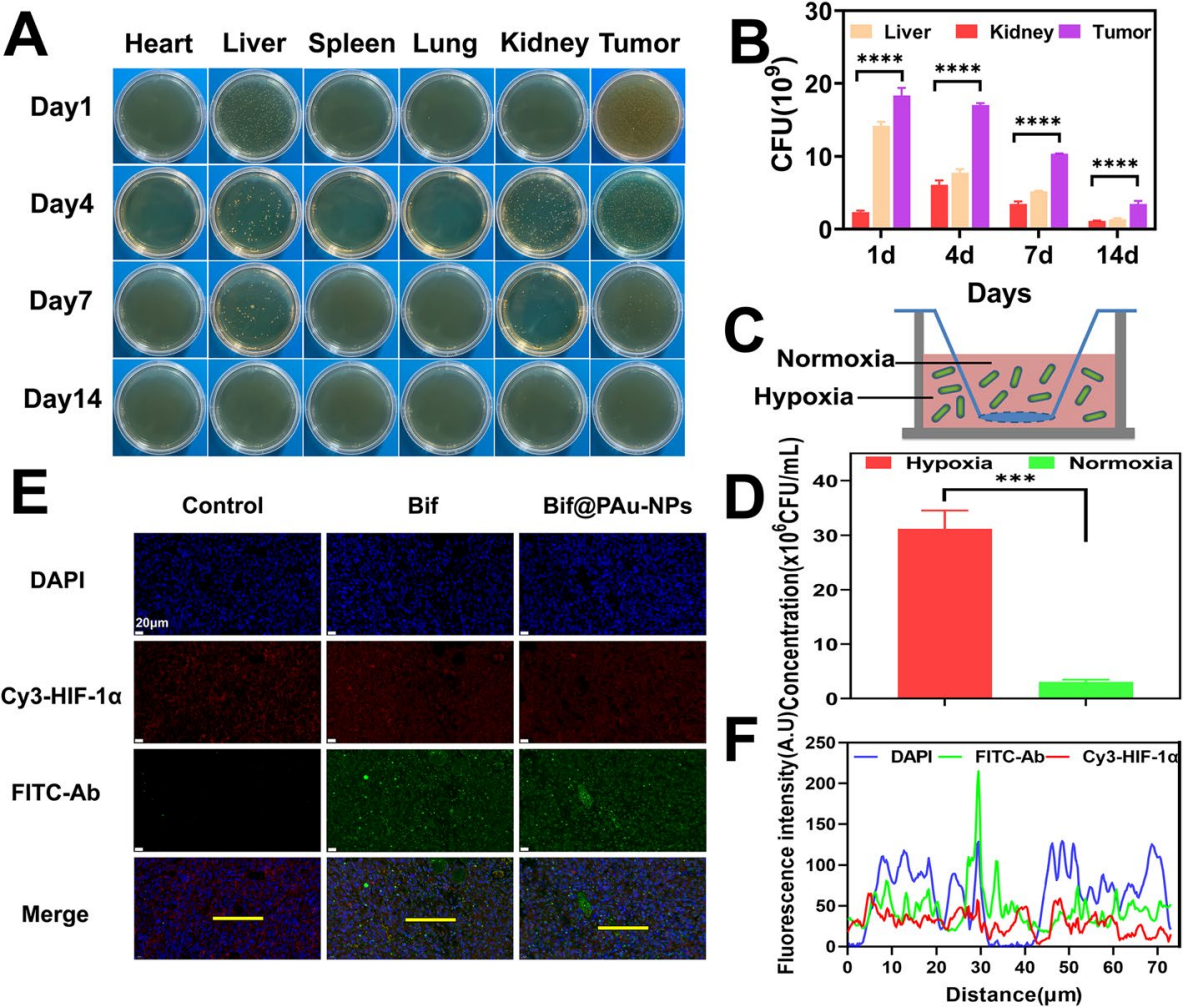
Targeting ability of Bif@PAu-NPs hybrids. **A** Representative photographs of bacterial growth in heart, liver, spleen, lung, kidney and tumor tissues on days 1, 4, 7 and 14 after Bif@PAu-NPs injection in CT-26 tumor-bearing mice. **B** Number of Bif@PAu-NPs colonization in liver, kidney and tumor. **C** Schematic diagram of in vitro hypoxia model using Transwell Chambers. **D** Bacteria number in Transwell chambers. **E** Fluorescence images of tumors. (Red: hypoxic zone, green: Bif, scale bar = 20 μm). **F** Fluorescence intensity of Bif@PAu-NPs and hypoxic zone along the yellow line). (***P < 0.001, ****P < 0.0001)

The selective accumulation of the Bif@PAu-NPs hybrids in the tumor tissues was verified through both in vitro and in vivo experiments. In the tumor-bearing mice, the bacteria were mainly distributed in the liver, kidneys, and tumor on day 1 and day 4. The bacterial load in the tumor tissues was significantly higher compared to other organs (*P* < 0.0001, Fig. 4A-B). In contrast, mice administrated with NS showed no bacterial growth (Figure S13). In the in vitro simulated hypoxic environment, the bacterial population in the anoxic chamber was significantly higher than that in the normoxic chamber (Fig. 4C-D). In mice injected with Bif@PAu-NPs, the FITC-stained Bif (green fluorescence) specifically co-localized with the Cy3-stained tumor hypoxic regions (red fluorescence, Fig. 4E). This result was confirmed by fluorescence intensity as well (Fig. 4F). In the NS group, only weak green fluorescence was observed (Figure S14), while the distribution of Bif was similar to that of Bif@PAu-NPs (Figure S15). This indicates that the Bif@PAu-NPs biohybrids retained the ability of Bif to preferentially colonize the oxygen-depleted zones. Additionally, the binding of PAu-NPs onto Bif did not affect bacterial growth (Figure S16).

### GM-CSF augmented the anti-tumor effects of Bif@PAu-NPs

**Fig. 5:**
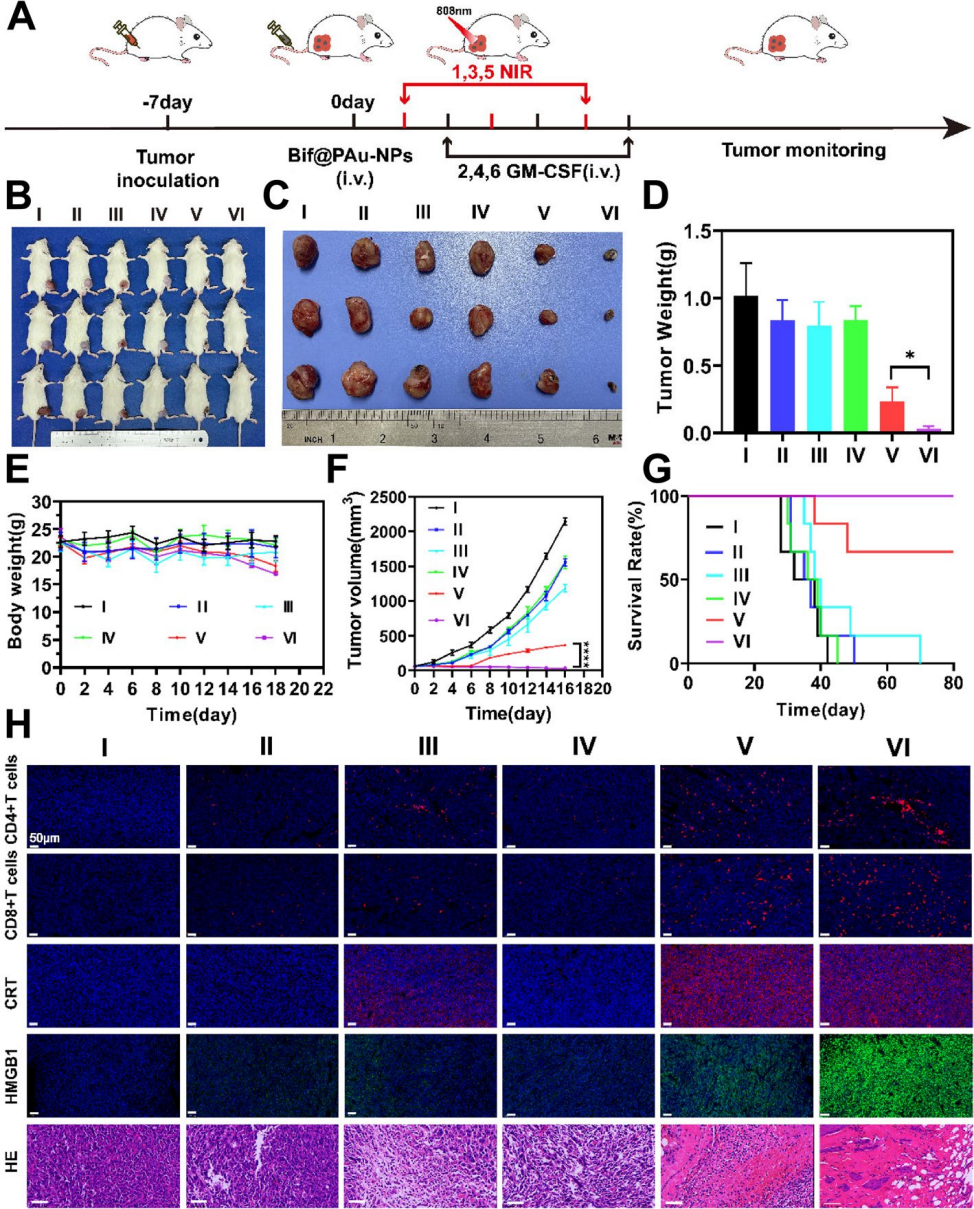
In vivo evaluation of antitumor efficacy. **A** Schematic diagram of the treatment procedure. **B** Representative photographs of CT-26 tumor-bearing mice on day 14 of post-treatment (*n* = 3). **C** Representative photographs of isolated tumors (*n* = 3). **D** Tumor weights. **E** Tumor volume (*n* = 6). **F** Change of body weight (*n* = 6). **G** Survival curves of mice (*n* = 6). **H** Immunofluorescence images of CD4, CD8, CRT and HMGB1, HE staining of tumors. scale bar = 50 μm. (*P < 0.05, ****P < 0.0001). I: Control; II: Bif + NIR + GM-CSF; III: PAu-NPs + NIR + GM-CSF; IV: Bif@PAu-NPs + GM-CSF; V: Bif@PAu-NPs + NIR; VI: Bif@PAu-NPs + NIR + GM-CSF

As shown in Fig. 5A, the CT-26 tumor-bearing mice were irradiated with a NIR laser to induce ICD. Subsequently, GM-CSF was administrated to recruit DCs and initiate T cell immunity. The tumor volume in the Bif@PAu-NPs + NIR + GM-CSF group (VI) was significantly lower (*P* < 0.0001) compared to that in the Bif@PAu-NPs + GM-CSF group (IV), indicating the effectiveness of PTT. Moreover, the tumor suppression rate in the Bif@PAu-NPs + NIR + GM-CSF group (VI) was 98.32% compared to 82.85% in the Bif@PAu-NPs + NIR group (V), indicating that inducing of DC-initiated adaptive immunity can enhance the anti-tumor effects of PTT (Fig. 5F). Consistent with this, the combination of Bif@PAu-NPs + NIR and GM-CSF significantly prolonged the survival of the tumor-bearing mice compared to the other groups, with all mice in this group being alive after 80 days of treatment (Fig. 5G). The weight of the tumors and their macroscopic images were in agreement with the results of tumor volume (Fig. 5B-D). To further investigate the mechanism of the anti-tumor immune response, we analyzed the percentage of CD8 + and CD4 + T cells in the tumor tissues of all groups. As shown in Fig. 5H, the tumors in the Bif@PAu-NPs + NIR group (V) and Bif@PAu-NPs + NIR + GM-CSF groups had greater infiltration of CD8 + and CD4 + T cells compared to the other groups. Additionally, Bif@PAu-NPs + NIR and Bif@PAu-NPs + NIR + GM-CSF significantly increased the levels of CRT and HMGB1 in the serum compared to the control group (Fig. 5H). Consistent with these findings, HE staining revealed significant tumor necrosis in the Bif@PAu-NPs + NIR and Bif@PAu-NPs + NIR + GM-CSF groups.

There was no significant change in the body weight of the mice in each group, indicating minimal systemic toxicity of Bif@PAu-NPs (Fig. 5E). Furthermore, none of the formulations (Bif, PAu-NPs, and Bif@PAu-NPs) caused significant hemolysis in vitro (Figure S17), as evidenced by microscopic images of erythrocytes (Figure S17A), hemolysis rates and UV-Vis absorption spectra (Figure S17A-D), thus indicating the excellent hemocompatibility of Bif@PAu-NPs. Additionally, mice injected with GM-CSF showed elevated WBC and NEU counts compared to the control group (Figure S18), while the biochemical indices remained within the normal range in all groups (Figure S18). The kidney and liver function indices were particularly normal in the Bif@PAu-NPs-treated group. Moreover, none of the vital organs (heart, liver, spleen, lung, kidney) exhibited any significant histological damage (Figure S19).

### Bif@PAu-NPs accumulated in the tumors and induced an adaptive immune response in the presence of GM-CSF

**Fig. 6:**
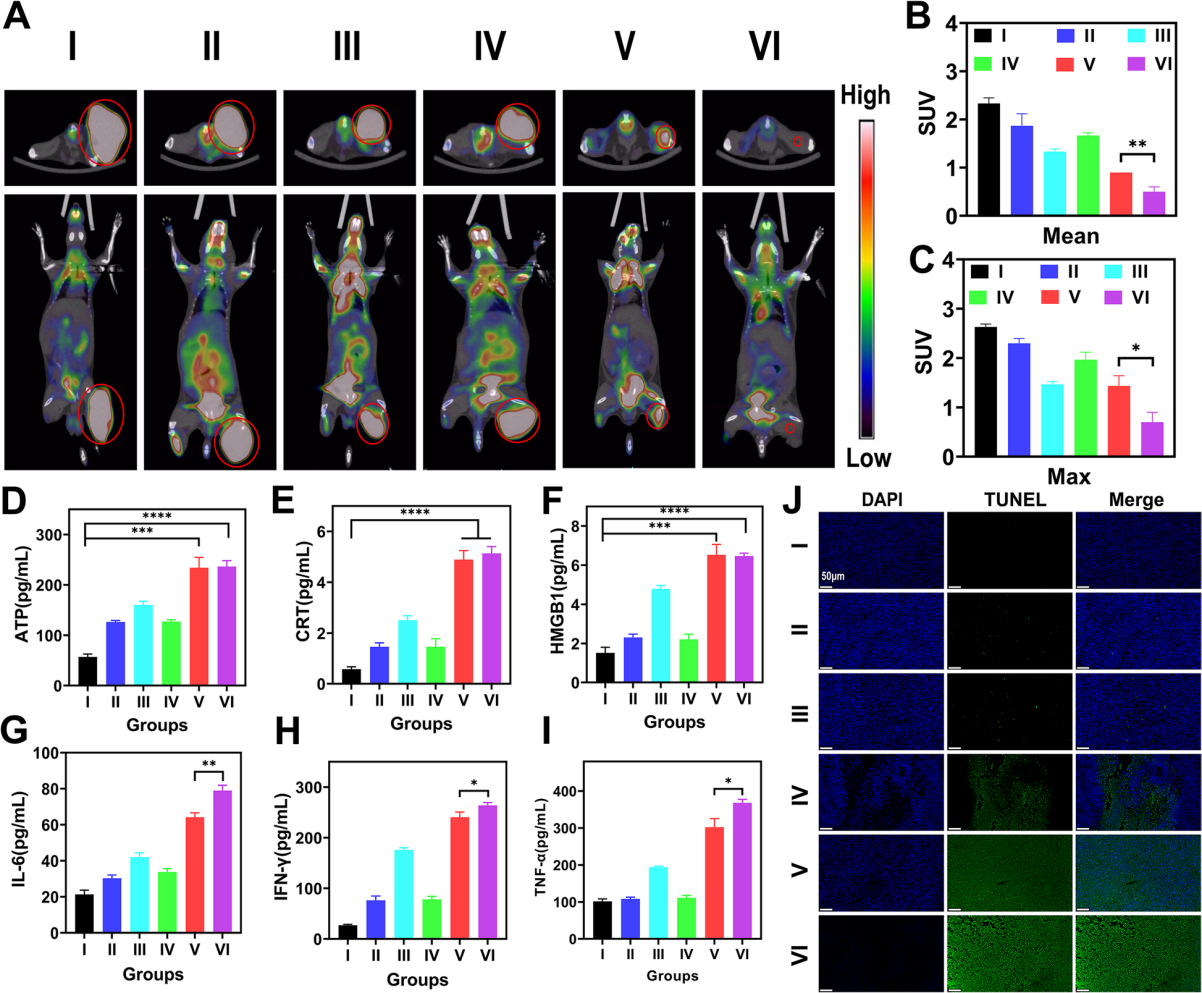
In vivo evaluation of early response to treatments and anti-tumor mechanism. **A** 18 F-FDG PET/CT images of mice in each group, upper: cross-sectional images, lower: coronal images, red circles indicate the tumor sites. SUVmean (**B**) and SUVmax (**C**) in each group. ATP secretion (**D**), CRT exposure (**E**) and the release of HMGB1(**F**) in tumor tissues detected by ELISA. Pro-inflammatory cytokine including IL-6 (**G**), IFN-γ (**H**) and TNF-α (**I**) analyzed by ELISA. Apoptosis of tumor tissues stained with DAPI and TUNEL(J), scale bar = 50 μm (*P < 0.05, **P < 0.01, ***P < 0.001, ****P < 0.0001). I: Control II: Bif + NIR + GM-CSF. III: PAu-NPs + NIR + GM-CSF. IV: Bif@PAu-NPs + GM-CSF. V: Bif@PAu-NPs + NIR. VI: Bif@PAu-NPs + NIR + GM-CSF.

Micro-PET/CT scan showed that the tumors in the Bif@PAu-NPs + NIR + GM-CSF group exhibited the lowest FDG uptake in both transverse and longitudinal views (Fig. 6A). This corresponded to the smallest maximum SUV and mean SUV values (Fig. 6B-C), indicating a significant decrease in metabolic activity in the tumors. Immunogenic cell death (ICD) initiates surface exposure of calreticulin (CRT), passive release of ATP and high mobility group box-1 (HMGB-1). After NIR laser irradiation, the levels of ATP (Fig. 6D), HMGB-1 (Fig. 6E), and CRT (Fig. 6F) in the serum increased significantly Bif@PAu-NPs + NIR and Bif@PAu-NPs + NIR + GM-CSF groups compared to the other groups. Additionally, the Bif@PAu-NPs + NIR + GM-CSF group exhibited the highest serum concentrations of IFN-γ, TNF-α and interleukin 6 (IL-6) (Fig. 6G-I), suggesting that PTT and GM-CSF could synergistically induce a robust immune response. This was further supported by the TUNEL staining of tumor tissues (Fig. 6J), which indicated that Bif@PAu-NPs + NIR + GM-CSF treatment significantly increased apoptosis rates and enhanced the anti-tumor effects.

**Fig. 7:**
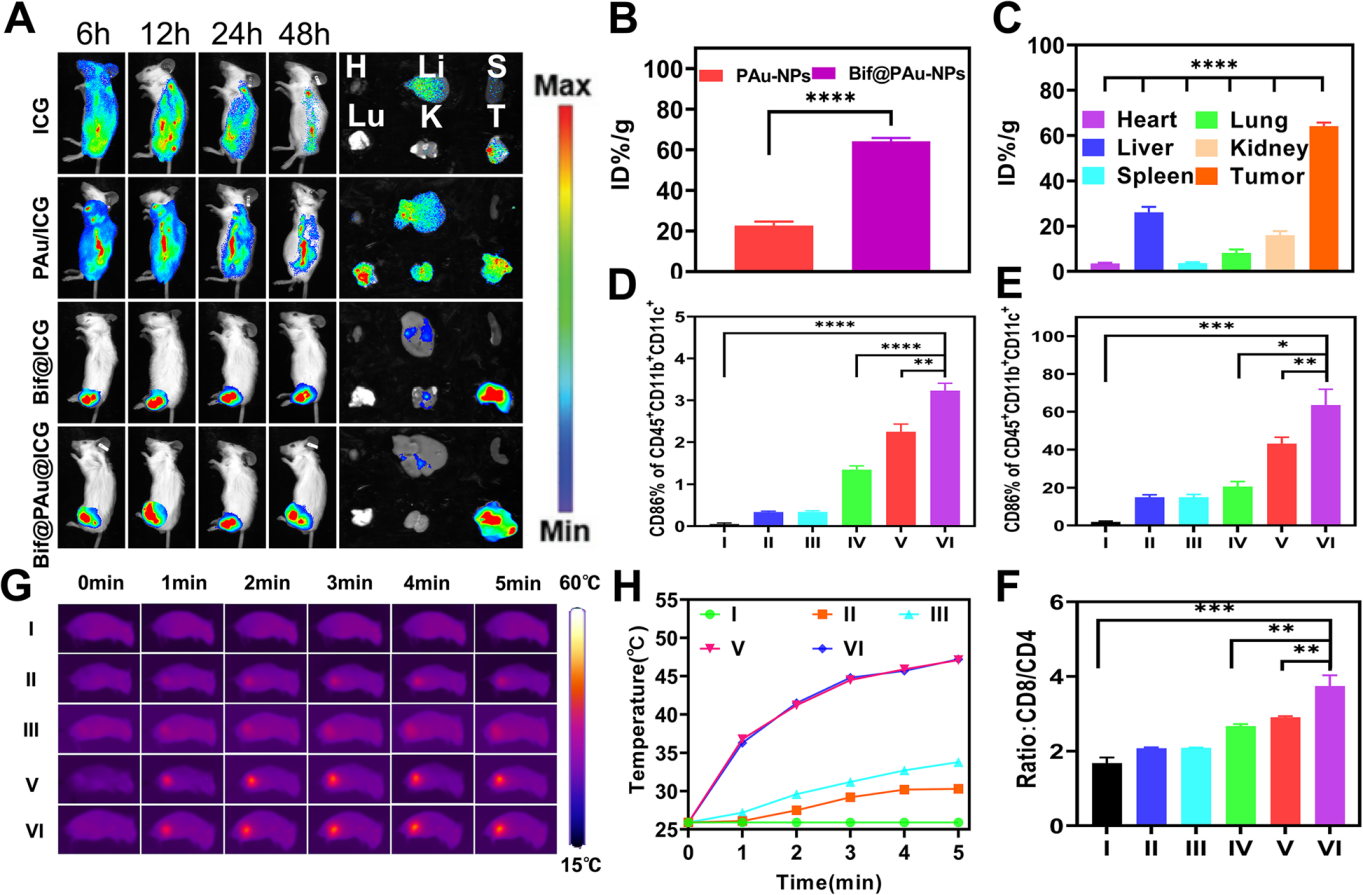
In vivo distribution the Bif@PAu-NPs hybrid and its anti-tumor immune response. **A** Fluorescence images of CT-26 tumor-bearing mice after intravenous injection of free ICG, PAu/ICG, ICG@Bif and Bif@PAu@ICG. The right panel shows in vitro fluorescence photos of isolated organs and tumors on 48 h after injection. (H: heart, Li: liver; S: spleen; Lu: lung, K: kidney, T: tumor). **B** Au content in tumors. **C** Au content in tumor and major organs of mice after Bif@PAu-NPs injection. **D** Percentage of mature DCs in spleen. **E** Percentage of mature DCs in tumor. **F** The ratio of CD8/CD4 in spleen. **G** Infrared thermography images of mice irradiated with NIR laser for 5 min. **H** The temperature change in each group (*P < 0.05, **P < 0.01, ***P < 0.001, ****P < 0.0001). Groups. I: Control. II: Bif + NIR + GM-CSF. III: PAu-NPs + NIR + GM-CSF. IV: Bif@PAu-NPs + GM-CSF. V: Bif@PAu-NPs + NIR. VI: Bif@PAu-NPs + NIR + GM-CSF.

The tumor targeting ability of Bif@PAu-NPs was also confirmed by tracking their biodistribution in vivo. As shown in Fig. 7A, the fluorescence intensity at the tumor sites of mice injected with ICG@Bif or Bif@PAu-NPs/ICG was significantly higher than that in the free ICG and PAu-NPs/ICG groups. This indicated that the Bif@PAu-NPs/ICG hybrid retained the active targeting ability of Bif. Additionally, strong fluorescent signals in the resected tumors also demonstrated the enhanced accumulation of Bif@PAu-NPs within the tumor tissues (Fig. 7A). ICP-MS further confirmed that the Au content was significantly higher in the Bif@PAu-NPs group compared to the other groups (*P* < 0.0001, Fig. 7B C), which was consistent with the tumor targeting ability of Bif@PAu-NPs. DCs are the most potent antigen-presenting cells that trigger antigen-specific T-cell responses. Enhanced infiltration of DCs into tumor tissues is essential for initiating anti-tumor immunity. We hypothesized that the increased recruitment of DCs by GM-CSF can activate T cells through the presentation of tumor-associated antigens released following PTT. Indeed, GM-CSF injection led to the maturation of DCs and their migration to peripheral immune organs compared to that in the NS group. Furthermore, the proportion of mature DCs (CD45 + CD11bCD11c + CD86+) in the spleen of the Bif@PAu-NPs + NIR + GM-CSF group was higher than that in the Bif@PAu-NPs + GM-CSF group (*P* < 0.0001). This indicated that PTT-induced ICD and GM-CSF can synergistically promote DCs recruitment and maturation (Fig. 7D, Figure S20). Similar trends were observed with the proportion of mature DCs in the tumor tissues (Fig. 7E, Figure S21). Additionally, the number of CD8 + cytotoxic T cells outnumbered the CD4 + helper T cells in the spleens of mice in the Bif@PAu-NPs + NIR + GM-CSF group by approximately 3.6-fold, compared to 1.6-fold in the NS group (Fig. 7F, Figure S22). The significant increase in the CD8/CD4 ratio suggested that Bif@PAu-NPs + NIR + GM-CSF can trigger an effective anti-tumor immune response. Furthermore, the temperature of the tumors treated with Bif@PAu-NPs rapidly increased to 47.2 °C under NIR laser irradiation (Fig. 7H), indicating that the active accumulation of self-driving Bif@PAu-NPs hybrids at the tumor site produced a photothermal effect. In contrast, PAu-NPs raised the intra-tumoral temperature to only about 33.8 °C, which can be attributed to the enhanced permeability and retention (EPR) effect. The thermal images of the mice treated with different formulations are shown in Fig. 7G.

###  Bif@PAu-NPs induced long-term immunological memory in combination with GM-CSF

**Fig. 8:**
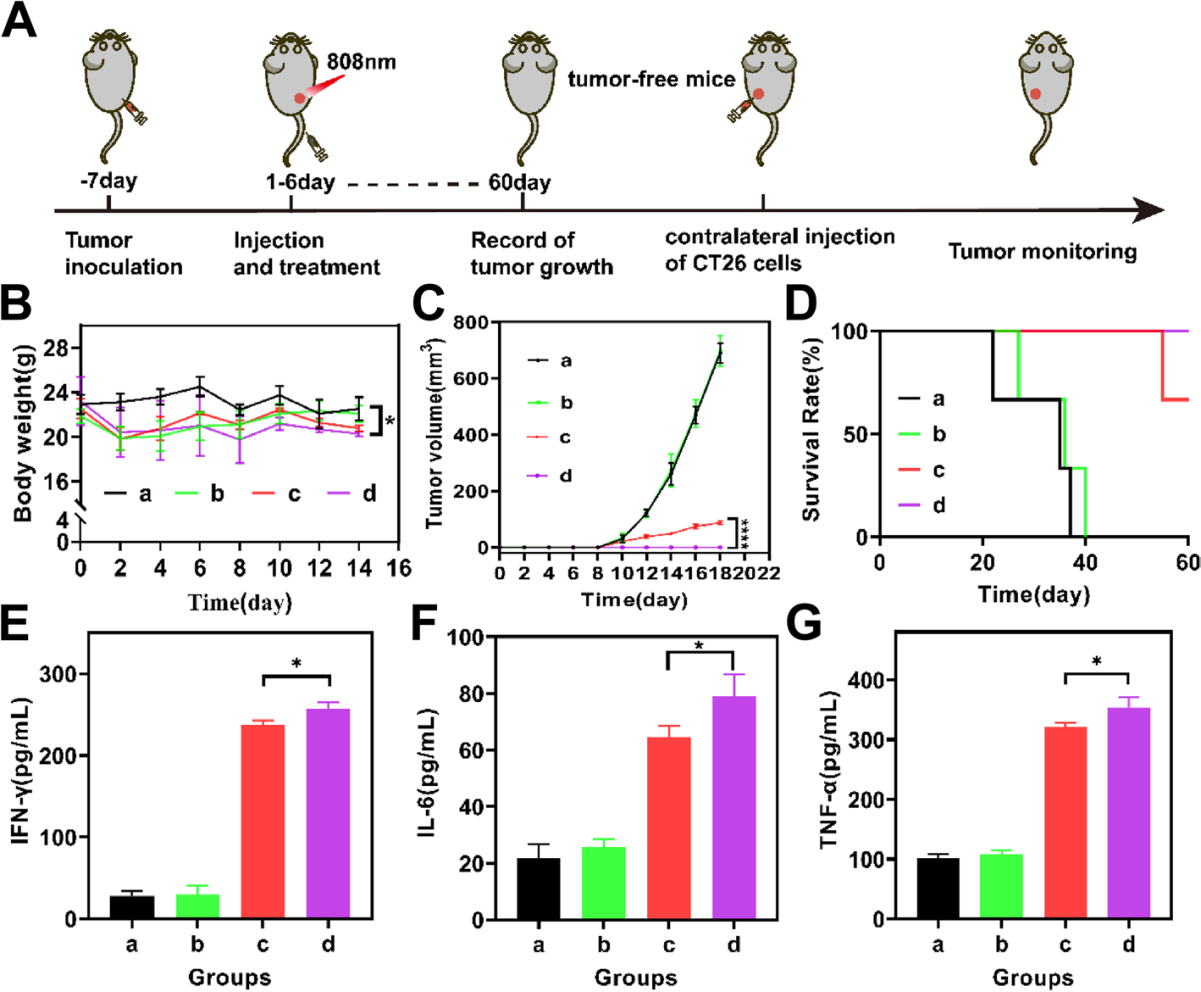
In vivo evaluation of long-term immunological memory by tumor rechallenge. **A** Schematic diagram of experimental design. **B** Weight fluctuations during treatment (*n* = 3). **C** Tumor volume of second tumors (*n* = 3). (D) Survival curves of mice (*n* = 6). Levels of pro-inflammatory cytokines including IFN-γ (**E**), IL-6 (**F**) and TNF-α (**G**). (*P < 0.05, ****P < 0.0001). a: Control, b: Bif@PAu-NPs, c: Bif@PAu-NPs + NIR, d: Bif@PAu-NPs + NIR + GM-CSF.

To determine whether the combination of GM-CSF and Bif@PAu-NPs-based PTT can induce long-term immune memory, we established secondary tumors as outlined in Fig. 8A. Briefly, the tumor-bearing mice were treated with NS, Bif@PAu-NPs, Bif@PAu-NPs + NIR and Bif@PAu-NPs + NIR + GM-CSF, and re-injected with CT-26 cells into their contralateral side 60 days after starting the treatment. The primary tumors in the Bif@PAu-NPs + NIR and Bif@PAu-NPs + NIR + GM-CSF groups underwent complete regression (CR). The body weight of mice in the Bif@PAu-NPs + NIR + GM-CSF group remained stable throughout the treatment, with little change compared to the control group (Fig. 8B). The mean volume of the secondary tumors in the control group exceeded 600 mm^3^, while it was only 87 mm^3^ in the Bif@PAu-NPs + NIR group 18 days post-inoculation. However, no secondary tumor growth was observed in the Bif@PAu-NPs + NIR + GM-CSF group (Fig. 8C), and the survival rate was 100% during the observation period (Fig. 8D). Thus, the combination of Bif@PAu-NPs-based PTT and GM-CSF elicited a strong immune memory that prevented tumor regrowth. Consistent with this, the levels of IFN-γ, TNF-α and IL-6 significantly increased in the serum of mice from the Bif@PAu-NPs + NIR + GM-CSF group compared to the other groups, indicating that a robust anti-tumor immune response triggered by the combination treatment (Fig. 8E-G). In conclusion, Bif@PAu-NPs hybrid-induced PTT in combination with GM-CSF can facilitate immune responses after eradicating the primary tumor and induce long-term immune memory to inhibit the growth of secondary tumor.

## Discussion

Nanoparticle-based PTT has received considerable attention in recent years [51] since it can avoid the risks associated with surgery and greatly reduces the cost of treatment. Gold nanoparticles (Au-NPs) have been utilized in tumor diagnosis, imaging, and targeted drug delivery, thanks to their ease of synthesis, and favorable physical, chemical, and optical properties [15, 52]. However, the challenge lies in effectively delivering photothermal agents to tumor tissues through active targeting, thus hindering the efficacy of Au-NPs-based PTT [53].

Anaerobic bacteria can actively deliver therapeutic agents to the hypoxic regions of solid tumors [27, 28]. For instance, there are reports of utilizing attenuated *Escherichia coli* to deliver the photosensitizer dihydroporphyrin e6 [29], and *Salmonella typhi* for targeted delivery of polydopamine [54]. In fact, *Salmonella* has been tested as a carrier for various photothermal and immunotherapeutic agents [30]. Consequently, anaerobic bacteria can be harnessed to deliver photothermal agents to the deep hypoxic regions of solid tumors. However, it is important to note that *E. coli* and *Salmonella* are pathogenic bacteria, so proper deactivation and engineering are necessary. This undoubtedly increases the technical challenges and costs. On the other hand, *B. infantis* (Bif) is an anaerobic probiotic bacteria that does not require deactivation or similar modifications [32]. Bif-based hybrids can selectively accumulate in the hypoxic zones of tumors due to the inherent tropism of the bacteria, resulting in targeted anti-tumor effects [33, 55]. Currently, NPs are conjugated to bacterial cells through chemical bonding [56], electrostatic adsorption [57], and antigen-antibody interactions [58]. In this study, we used PDA as a linker to bind Au-NPs to Bif because of its advantages in photothermal conversion, adhesion, good biocompatibility and non-toxicity [20, 22, 25]. After PDA decoration, the PAu-NPs not only exhibited low cytotoxicity in vitro but also retained the targeting ability of Bif to hypoxic regions.

The Bif@PAu-NPs hybrids also retained the photothermal properties of PAu-NPs, as well as the biological activity of Bif. These hybrids actively accumulated in the hypoxic zones of the tumor in vivo, validating Bif as a reliable carrier for Au-NPs. Moreover, PTT based on Bif@PAu-NPs triggered the release of DAMPs from the dying tumor cells, which in turn initiated an adaptive immune response. The presence of GM-CSF further promoted DCs maturation and T cells activation, not only enhancing PTT-induced immune responses but also inducing long-term immune memory. Additionally, even at high concentrations, Bif@PAu-NPs were non-toxic in the absence of NIR laser irradiation, demonstrating the safety of this formulation for clinical applications. As Bif@PAu-NPs did not induce hemolysis, they can be administered intravenously. The bacterial hybrids exhibited significant tumor regression and prolonged survival, particularly, in the presence of GM-CSF, demonstrating their anti-tumor effects and biosafety.

In summary, the biohybrids Bif@PAu-NPs can effectively deliver Au-NPs to the hypoxic zones of solid tumors and improve the targeted delivery of photothermal agents. Surface modification with PDA not only allows the Au-NPS to adhere to the bacterial cells, but also enhances their photothermal effects. Furthermore, as an immune adjuvant [47], GM-CSF augmented the PTT-induced immune response to establish long-term immune memory. Thus, Bif@PAu-NPs biohybrids may be a promising tool for combining PTT with chemotherapy or immunotherapy. However, satisfactory immunotherapeutic outcomes depend not only on effective drug delivery but also on the tumor microenvironment. Immunologically ‘cold’ tumors are insensitive to immune checkpoint inhibitors due to their low immunogenicity, which may impair the activity of tumor-specific cytotoxic T cells [59]. Additionally, PTT can rapidly induce the production of heat shock protein (HSP), a key molecular chaperone protein that enhances thermotolerance, leading to a decrease in PTT efficiency [60]. Therefore, future studies should focus on reducing heat resistance in vivo to optimize the efficacy of PTT.

## Conclusion

We developed a bioactive hybrid Bif@PAu-NPs for targeted therapy against CRC. The PDA-coated Au-NPs were attached to the surface of the anaerobic *B. infantis*. Due to the natural affinity of Bifidobacterium infantis to hypoxic regions, the Bif@PAu-NPs hybrid selectively accumulated at the tumor site. When exposed to 808 nm NIR laser irradiation, the hybrid produced local hyperthermia. This resulted in immunogenic cell death, leading to the release of tumor-associated antigens and DAMPs. Additionally, GM-CSF enhanced the recruitment of DCs to the tumors, boosting the immune responses induced by PTT. These combined effected resulted in significant anti-tumor effects and immune memory against tumor metastasis and recurrence. Overall, our study presents an innovative strategy for synergistic and integrative cancer therapy.

### Supplementary Information

**Additional file 1: Figure S1.** SEM image of Bif@PAu-NPs **Figure S2.** Zeta potential of Au-NPs and PAu-NPs. **Figure S3.** Energy spectrum of Bif@PAu-NPs. (A) EDS measurement, (B) elemental mapping, (C) total spectrum of elemental distribution (C, N, O and Au). **Figure S4. **XRD patterns of Au-NPs. **Figure S5. **In vitro stability analysis of the Bif@PAu-NPs biohybrids. (A) the appearance of the Bif@PAu-NPs biohybrids incubated in different solutions for 4 hours (left tube: pH=7.4, 0 mM GSH; right tube: pH=6.5, 10 mM GSH). (B) UV-vis absorbance curves of supernatants after centrifugation. **Figure S6.** UV-Vis-NIR absorption spectra of PAu-NPs at different concentrations. **Figure S7.** Color of PAu-NPs solutions with different concentrations. **Figure S8.** Infrared thermal images of PAu-NPs at different powers under 808 nm laser irradiation. **Figure S9. **Apoptosis rate of AML12 cells after different treatments. **Figure S10.** Live/dead staining of A549 cells after different treatments. (green, live cells) and (red, dead cells). Scale bar = 500 μm. **Figure S11.** Live/dead staining of 4T1 cells after different treatments. (green, live cells) and (red, dead cells). Scale bar = 500 μm. **Figure S12.** Flow cytometry analysis of CT26 cells after treatment for 24 hours. I: Control; II: Au-NPs; III: PAu-NPs; IV: Bif@PAu-NPs; V: NIR; VI: Au-NPs+NIR; VII: PAu-NPs+NIR; VIII: Bif@PAu-NPs+NIR. **Figure S13.** Bacterial growth in main organs and tumor on days 1, 4, 7 and 14 after injection of Bif@PAu-NPs in CT26 tumor-bearing mice. **Figure S14.** Fluorescence intensity along the yellow line in the control group (NS) shown in (Fig. 4E). **Figure S15.** Fluorescence intensity along the yellow line in the Bif group shown in Fig. 4E. **Figure S16. **The photo and bacteria number of Bif alone and Bif@PAu-NPs after 24 h of anaerobic incubation (*n*=3). ns: no significance. **Figure S17.** In vitro hemolysis analysis. (A) Representative micrographs of erythrocytes cultured with different drugs. a: normal saline (NS, negative control); b: distilled water (DW, positive control); c: Bif; d: PAu-NPs; e: Bif@PAu-NPs; B. Photographs of hemolysis test. (C) Hemolysis rate. (D) UV-vis absorption spectra of each group. **Figure S18. **In vivoevaluation of systemictoxicity by analyzing biochemical markers and routine blood indicators. **Figure S19.** H&E staining of major organs (heart, liver, spleen, lung and kidney) after different treatments. Scale bar=50 μm. Groups. I: Control. II: Bif+NIR+GM-CSF. III: PAu-NPs+NIR+GM-CSF. IV: Bif@PAu-NPs+GM-CSF. V: Bif@PAu-NPs+NIR. VI: Bif@PAu-NPs+NIR+GM-CSF. **Figure S20.** Flow cytometry analysis of CD86 (CD45+CD11b+CD11c+CD86+ as marker) in spleens after different treatments. **Figure S21.** Flow cytometry analysis of CD86 (CD45+CD11b+CD11c+CD86+ as marker) in tumor tissues after different treatments. **Figure S22.** Flow cytometry analysis of CD4+ (CD3+CD4+ as marker) and CD8+ (CD3+CD8+ as marker) T cell populations in the spleens after different treatments.

## Data Availability

All data associated with this study are present in the paper or the Supplementary information. All relevant data are available from the authors.
