# Supplementary material for: GM-CSF augmented the photothermal immunotherapeutic outcome of self-driving gold nanoparticles against a mouse CT-26 colon tumor model
Source: Biomater Res. 2023 Oct 23;27:105. doi: 10.1186/s40824-023-00430-6 (PMC10594909; doi:10.1186/s40824-023-00430-6)
Supplement: Supplementary file 1 — Additional file 1: Figure S1. SEM image of Bif@PAu-NPs Figure S2. Zeta potential of Au-NPs and PAu-NPs. Figure S3. Energy spectrum of Bif@PAu-NPs. (A) EDS measurement, (B) elemental mapping, (C) total spectrum of elemental distribution (C, N, O and Au). Figure S4. XRD patterns of Au-NPs. Figure S5. In vitro stability analysis of the Bif@PAu-NPs biohybrids. (A) the appearance of the Bif@PAu-NPs biohybrids incubated in different solutions for 4 hours (left tube: pH=7.4, 0 mM GSH; right tube: pH=6.5, 10 mM GSH). (B) UV-vis absorbance curves of supernatants after centrifugation. Figure S6. UV-Vis-NIR absorption spectra of PAu-NPs at different concentrations. Figure S7. Color of PAu-NPs solutions with different concentrations. Figure S8. Infrared thermal images of PAu-NPs at different powers under 808 nm laser irradiation. Figure S9. Apoptosis rate of AML12 cells after different treatments. Figure S10. Live/dead staining of A549 cells after different treatments. (green, live cells) and (red, dead cells). Scale bar = 500 μm. Figure S11. Live/dead staining of 4T1 cells after different treatments. (green, live cells) and (red, dead cells). Scale bar = 500 μm. Figure S12. Flow cytometry analysis of CT26 cells after treatment for 24 hours. I: Control; II: Au-NPs; III: PAu-NPs; IV: Bif@PAu-NPs; V: NIR; VI: Au-NPs+NIR; VII: PAu-NPs+NIR; VIII: Bif@PAu-NPs+NIR. Figure S13. Bacterial growth in main organs and tumor on days 1, 4, 7 and 14 after injection of Bif@PAu-NPs in CT26 tumor-bearing mice. Figure S14. Fluorescence intensity along the yellow line in the control group (NS) shown in (Fig. 4E). Figure S15. Fluorescence intensity along the yellow line in the Bif group shown in Fig. 4E. Figure S16. The photo and bacteria number of Bif alone and Bif@PAu-NPs after 24 h of anaerobic incubation (n=3). ns: no significance. Figure S17. In vitro hemolysis analysis. (A) Representative micrographs of erythrocytes cultured with different drugs. a: normal saline (NS, negative control); b: di [file 40824_2023_430_MOESM1_ESM.docx]

**Supplementary Materials**

**GM-CSF augmented the photothermal immunotherapeutic outcome of self-driving gold nanoparticles against a mouse CT-26 colon tumor model**

Jie Dai^a,#^, Jianmei Li^a,#^, Yuqin Zhang ^a,#^, Qian Wen^a^, Yun Lu^a^, Yu Fan^a^, Fancai Zeng^b^, Zhiyong Qian^c^, Yan Zhang^d*^, Shaozhi Fu^a,e*^


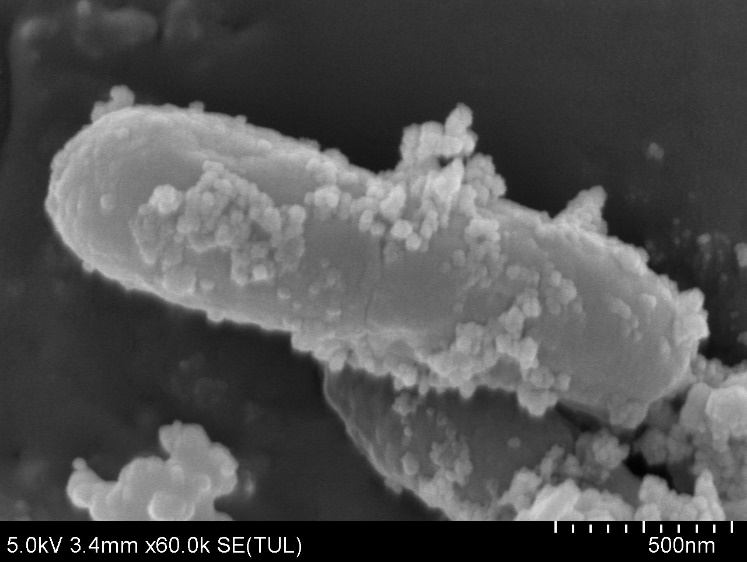


**Figure S1.** SEM image of Bif@PAu-NPs


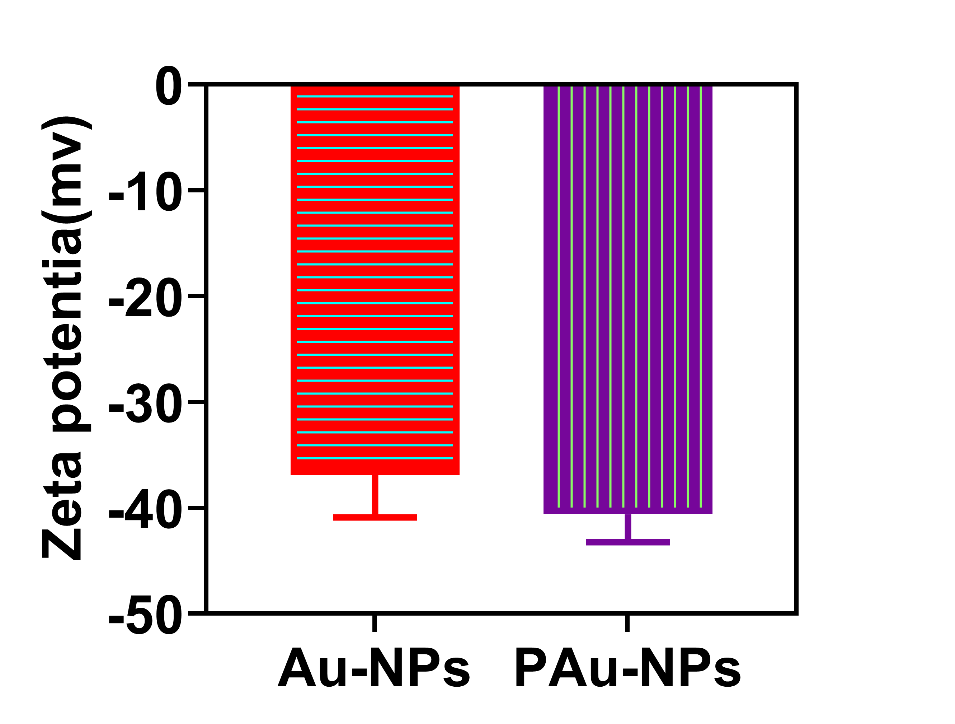


**Figure S2.** Zeta potential of Au-NPs and PAu-NPs.


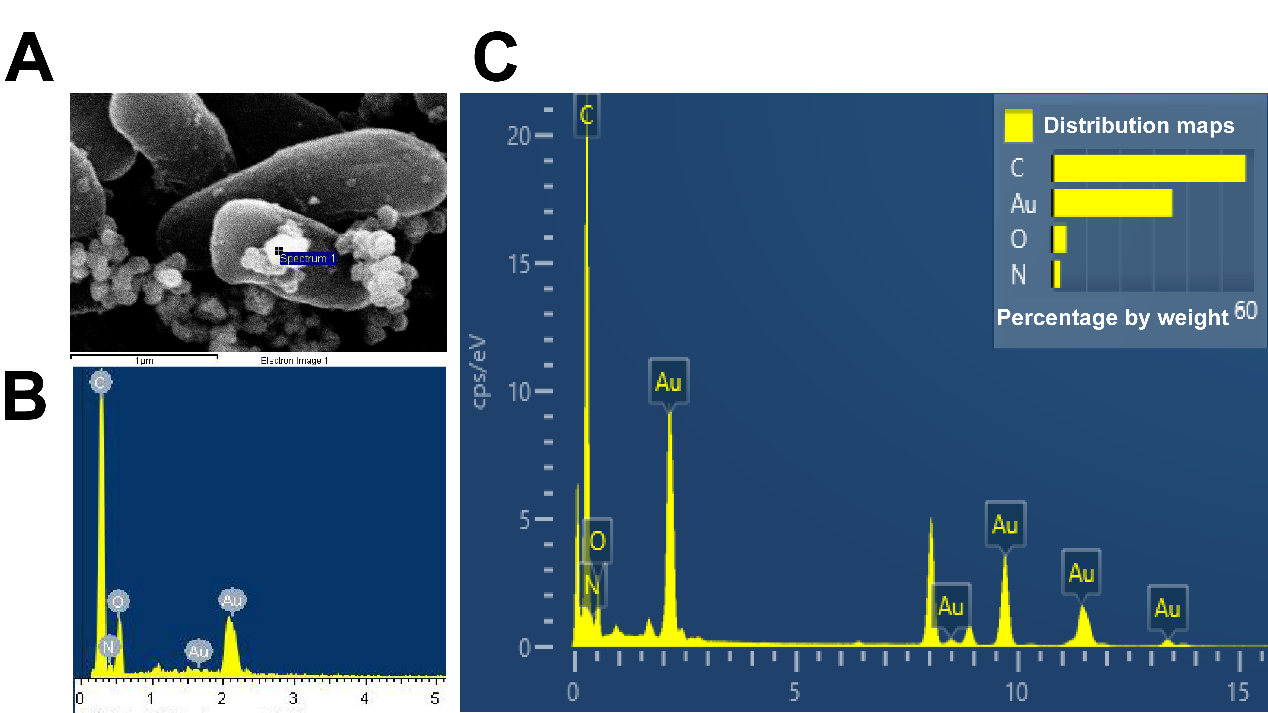


**Figure S3.** Energy spectrum of Bif@PAu-NPs. (A) EDS measurement, (B) elemental mapping, (C) total spectrum of elemental distribution (C, N, O and Au).


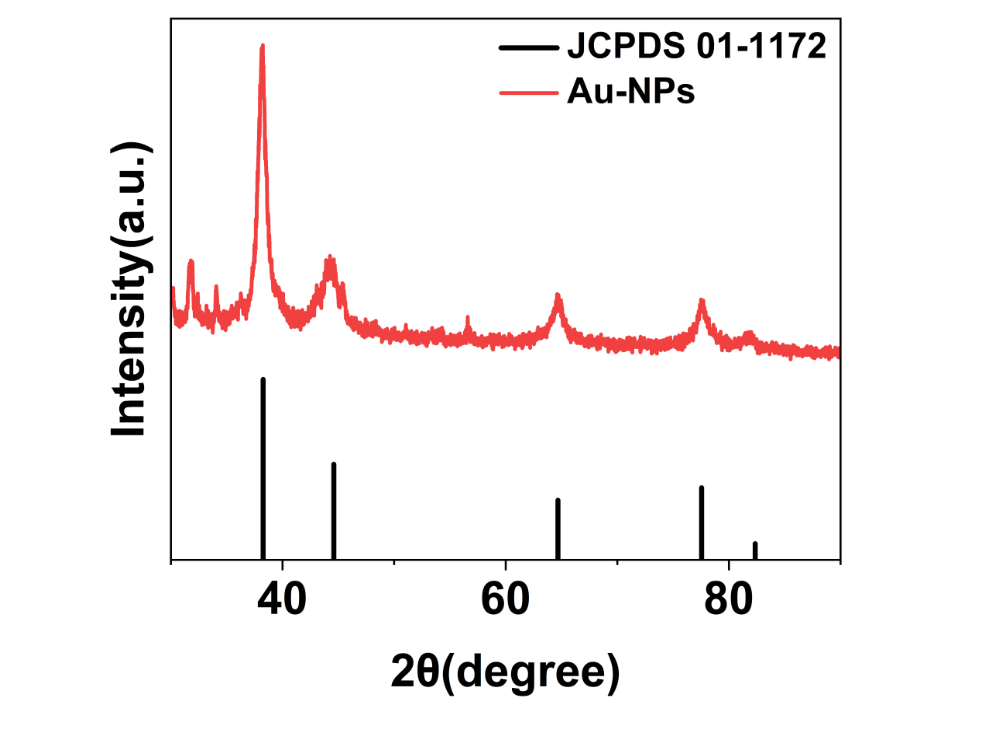


**Figure S4.** XRD patterns of Au-NPs.


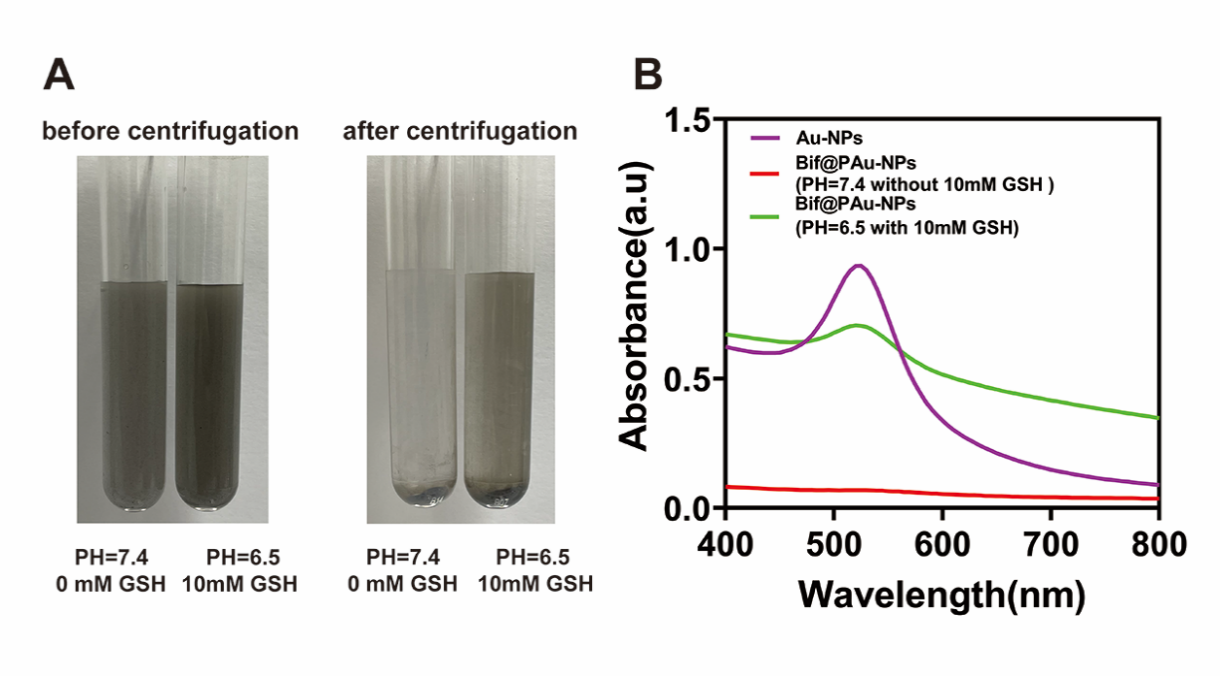


**Figure S5.** In vitro stability analysis of the Bif@PAu-NPs biohybrids. (A) the appearance of the Bif@PAu-NPs biohybrids incubated in different solutions for 4 hours (left tube: pH=7.4, 0 mM GSH; right tube: pH=6.5, 10 mM GSH). (B) UV-vis absorbance curves of supernatants after centrifugation.


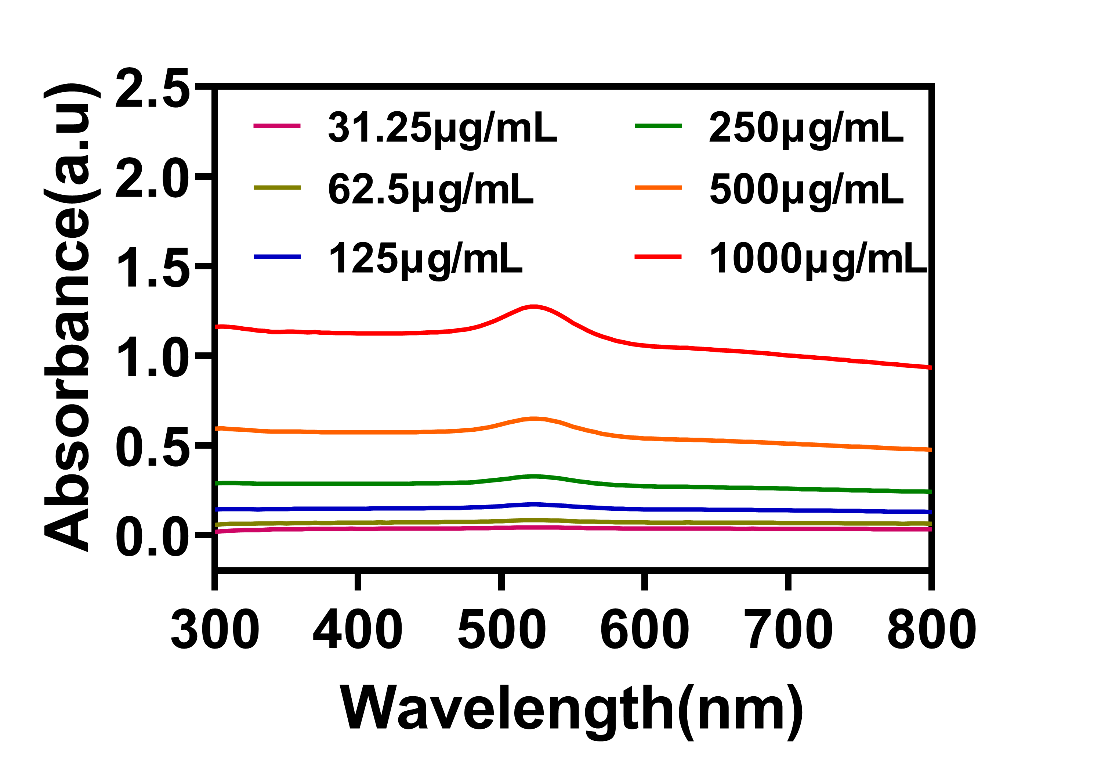


**Figure S6.** UV-Vis-NIR absorption spectra of PAu-NPs at different concentrations.


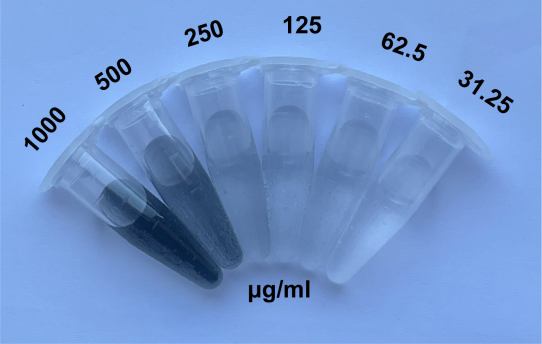


**Figure S7.** Color of PAu-NPs solutions with different concentrations.


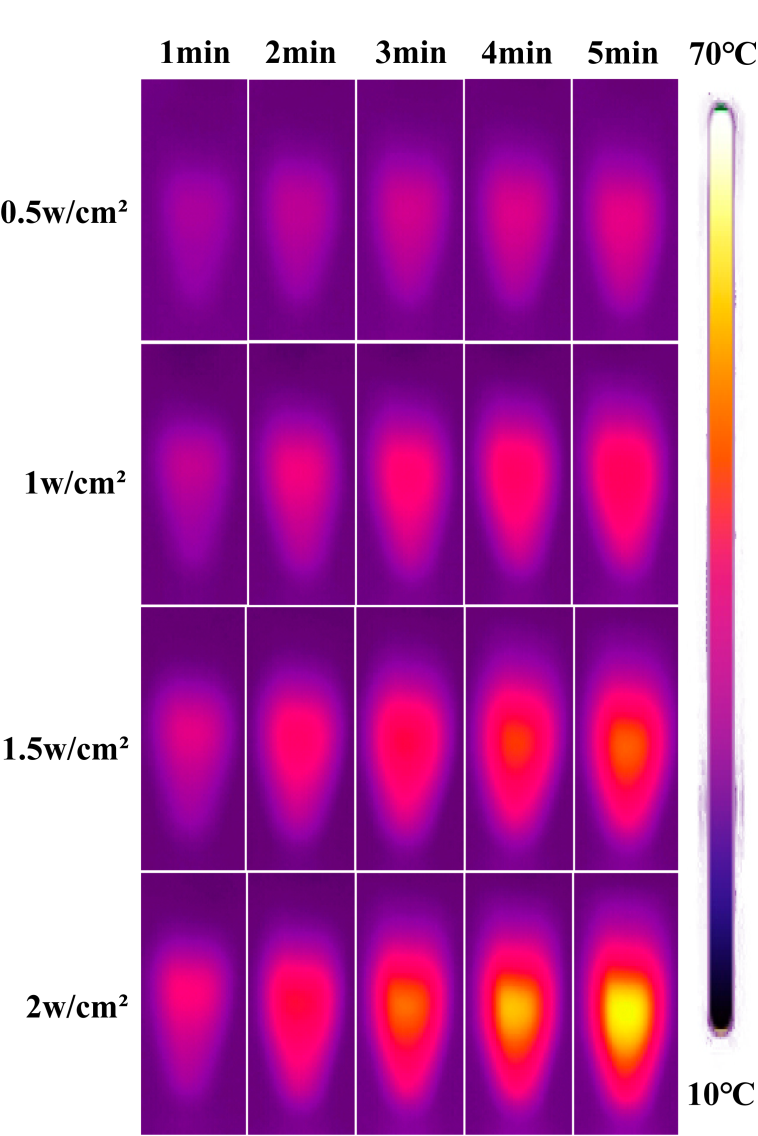


**Figure S8.** Infrared thermal images of PAu-NPs at different powers under 808 nm laser irradiation.

**
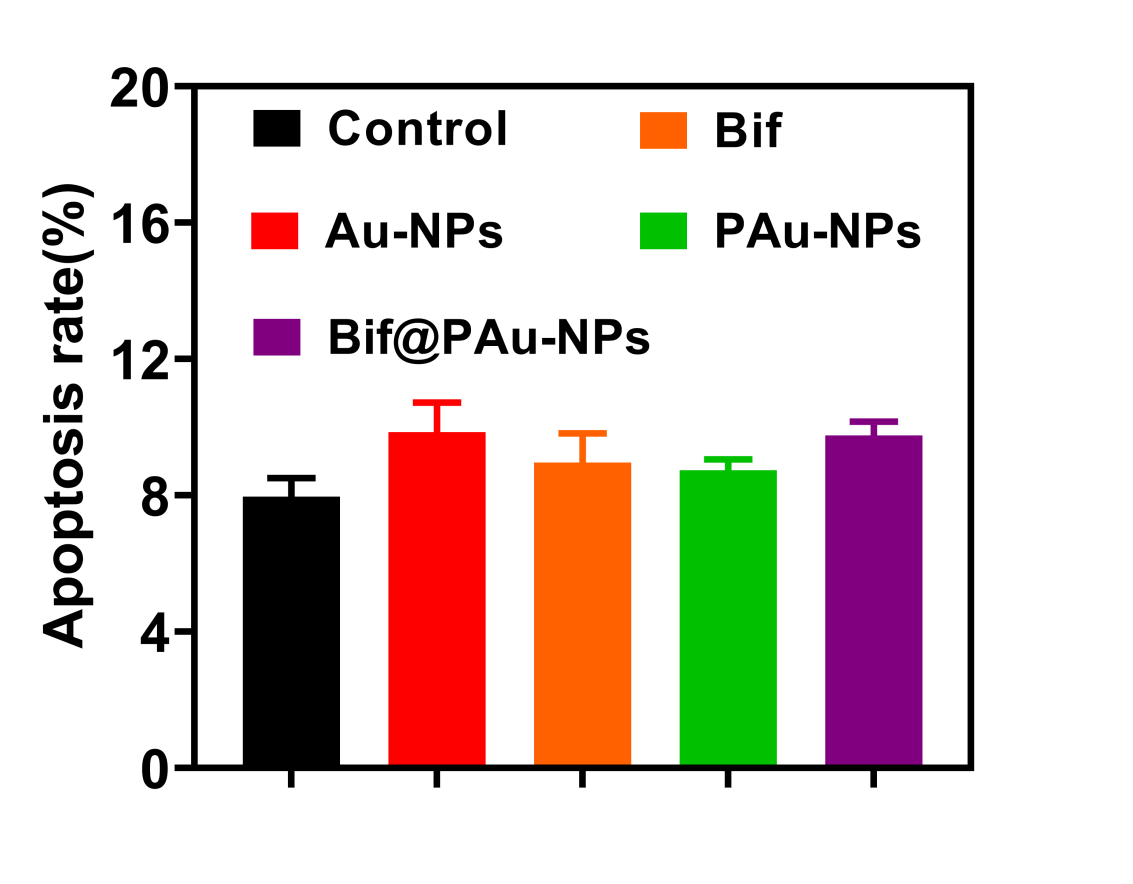
**

**Figure S9.** Apoptosis rate of AML12 cells after different treatments.


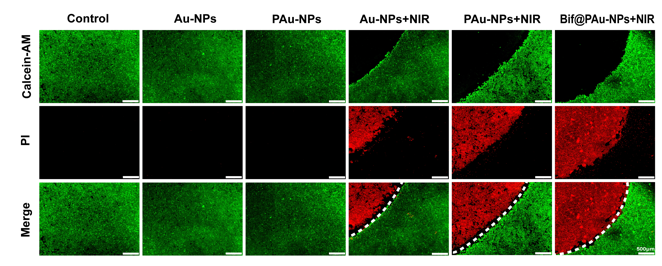


**Figure S10.** Live/dead staining of A549 cells after different treatments. (green, live cells) and (red, dead cells). Scale bar = 500 μm.


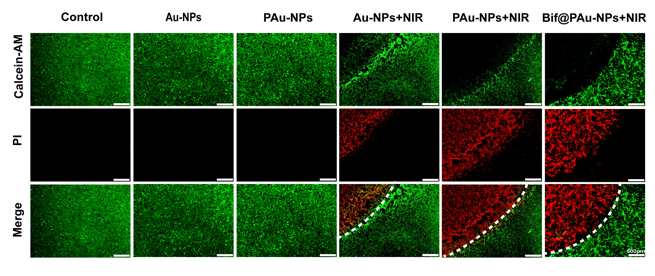


**Figure S11.** Live/dead staining of 4T1 cells after different treatments. (green, live cells) and (red, dead cells). Scale bar = 500 μm.

**
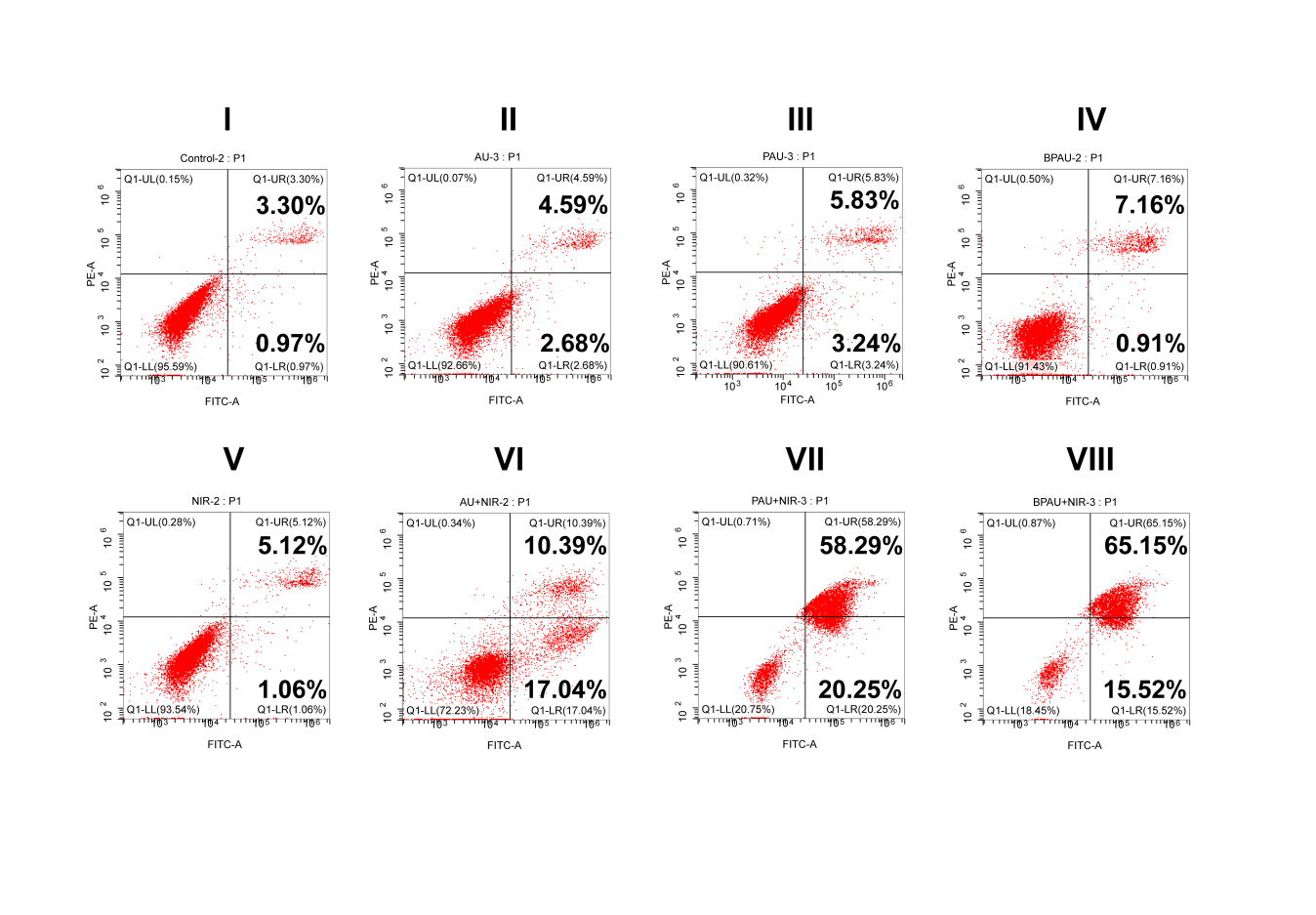
**

**Figure S12.** Flow cytometry analysis of CT26 cells after treatment for 24 hours. I: Control；II: Au-NPs; III: PAu-NPs; IV: Bif@PAu-NPs; V: NIR；VI: Au-NPs+NIR; VII: PAu-NPs+NIR; VIII: Bif@PAu-NPs+NIR.

**
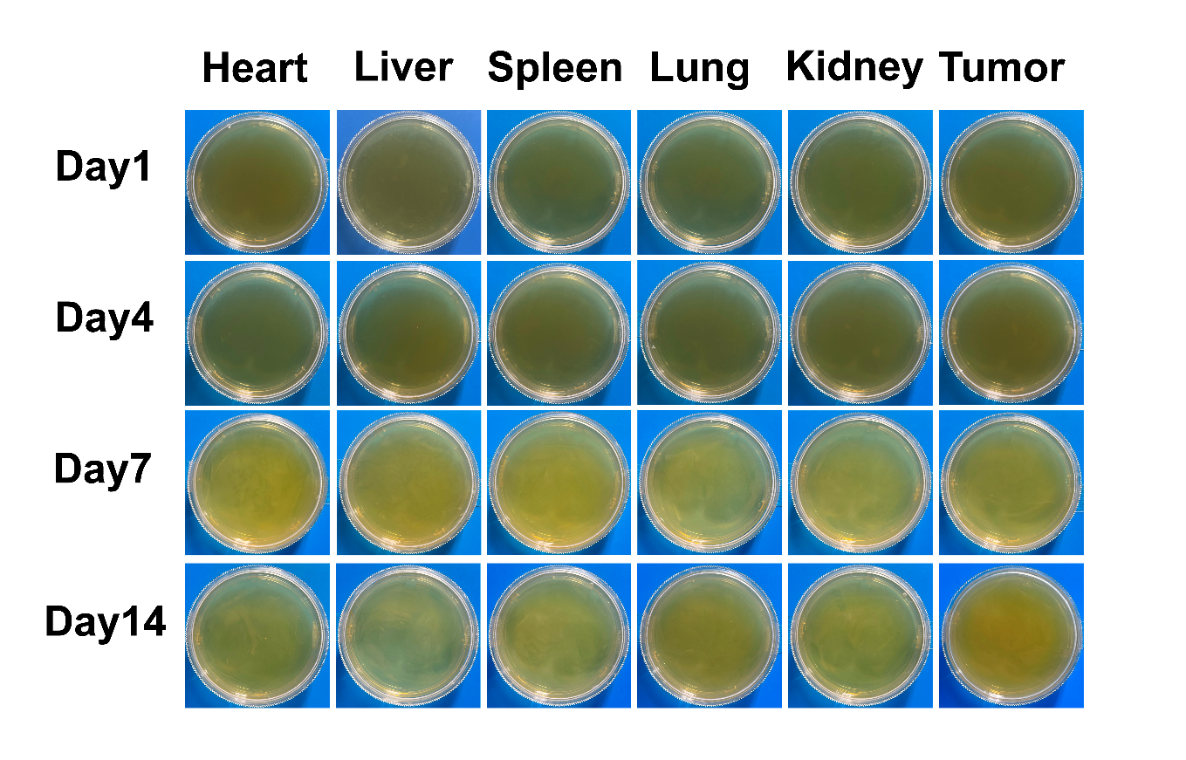
**

**Figure S13.** Bacterial growth in main organs and tumor on days 1, 4, 7 and 14 after injection of Bif@PAu-NPs in CT26 tumor-bearing mice.

**
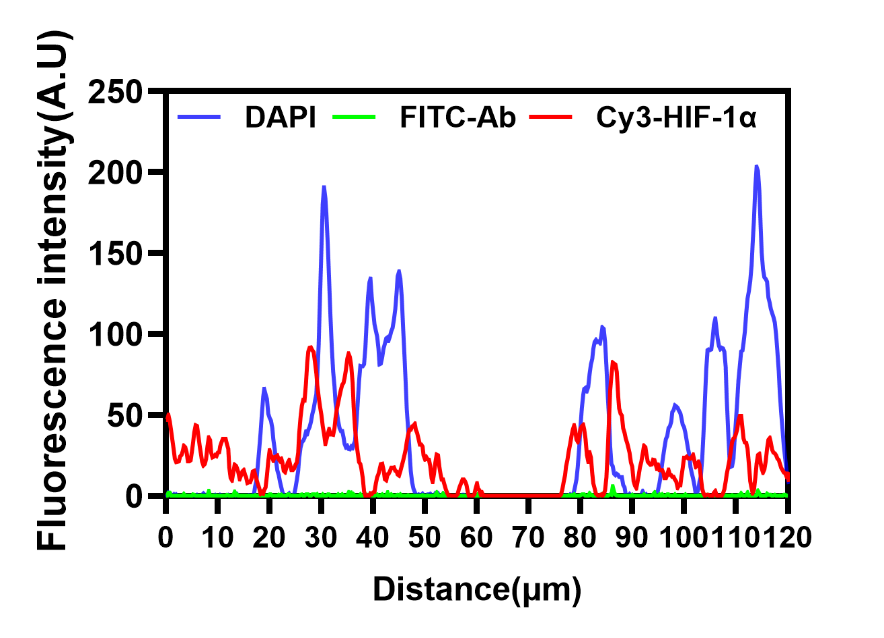
**

**Figure S14.** Fluorescence intensity along the yellow line in the control group (NS) shown in (Figure 4E).

**
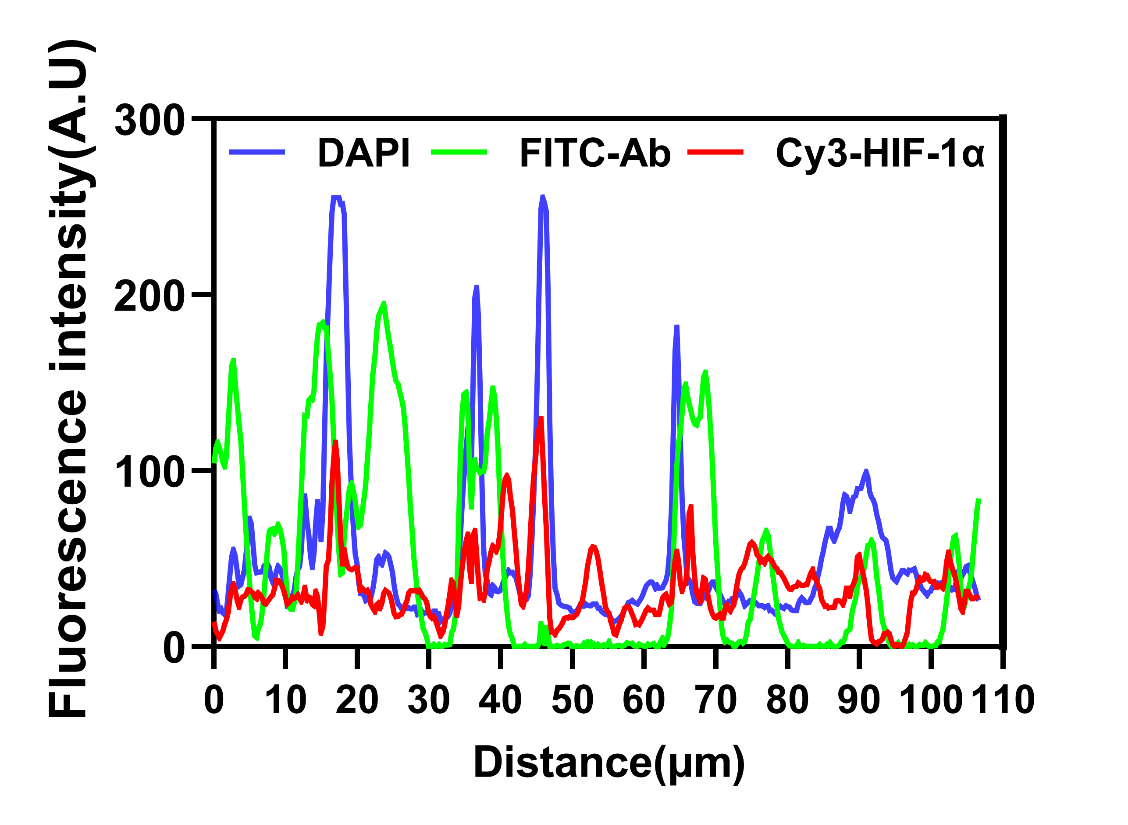
**

**Figure S15.** Fluorescence intensity along the yellow line in the Bif group shown in Figure 4E.

**
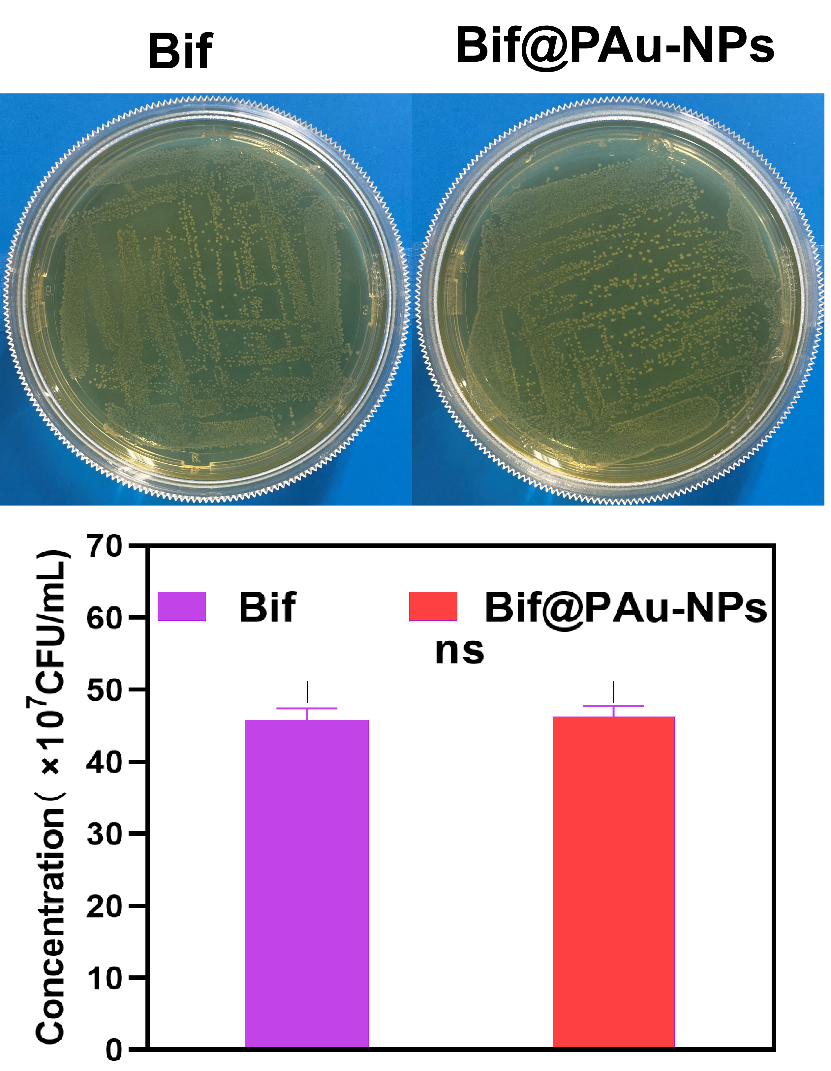
**

**Figure S16.** The photo and bacteria number of Bif alone and Bif@PAu-NPs after 24 h of anaerobic incubation (n=3). ns: no significance.

**
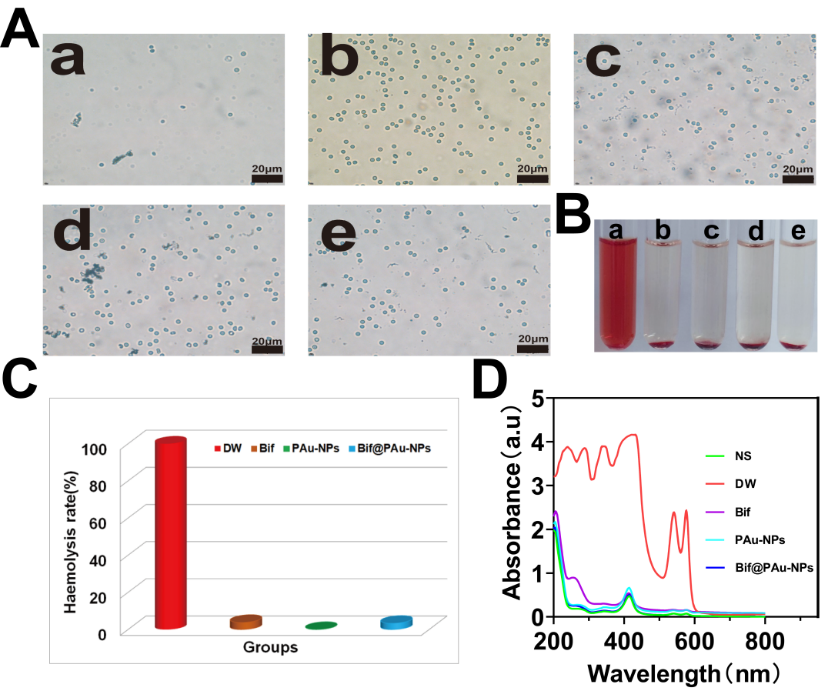
**

**Figure S17.** In vitro hemolysis analysis. (A) Representative micrographs of erythrocytes cultured with different drugs. a: normal saline (NS, negative control); b: distilled water (DW, positive control); c: Bif; d: PAu-NPs; e: Bif@PAu-NPs; B. Photographs of hemolysis test. (C) Hemolysis rate. (D) UV-vis absorption spectra of each group.

**
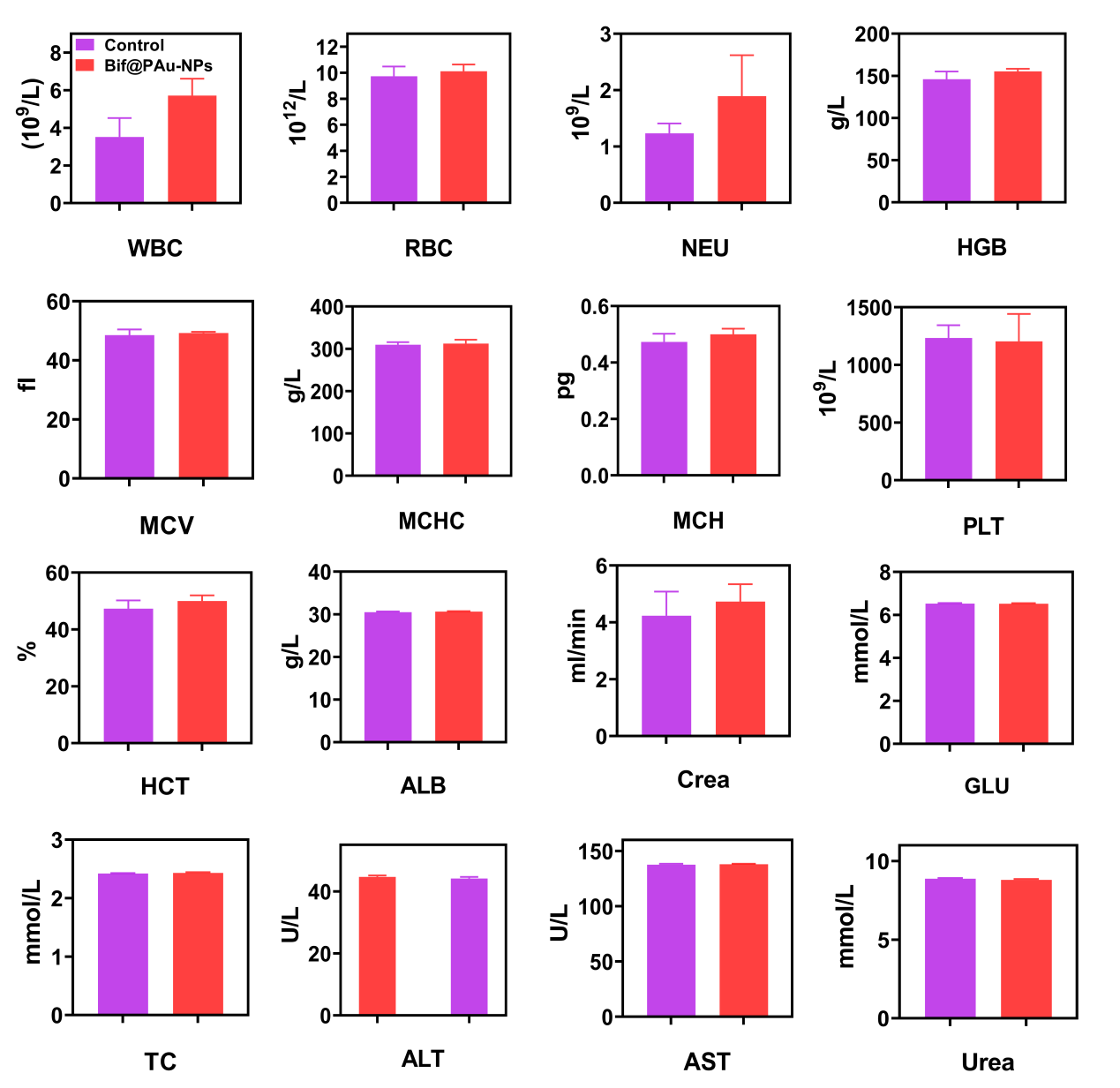
**

**Figure S18.** *In vivo* evaluation of systemic toxicity by analyzing biochemical markers and routine blood indicators.

**
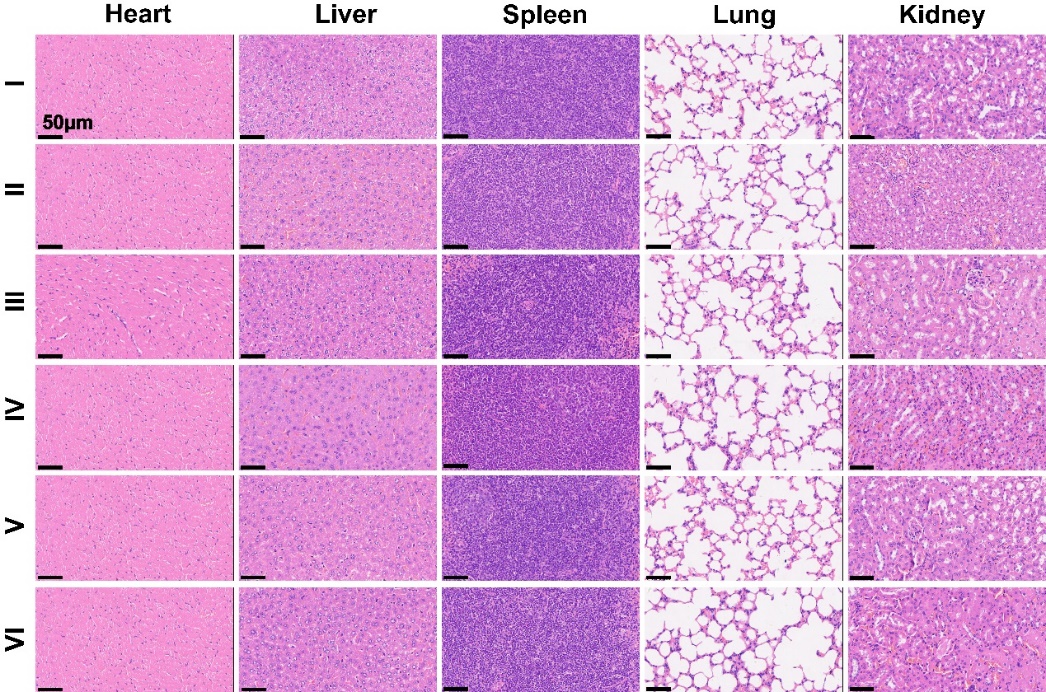
**

**Figure S19.** H&E staining of major organs (heart, liver, spleen, lung and kidney) after different treatments. Scale bar=50 μm. Groups. I: Control. II: Bif+NIR+GM-CSF. III: PAu-NPs+NIR+GM-CSF. IV: [Bif@PAu-NPs+GM-CSF. V: Bif@PAu-NPs+NIR](mailto:Bif@PAu-NPs+GM-CSF.V:Bif@PAu-NPs+NIR). VI: Bif@PAu-NPs+NIR+GM-CSF.

**
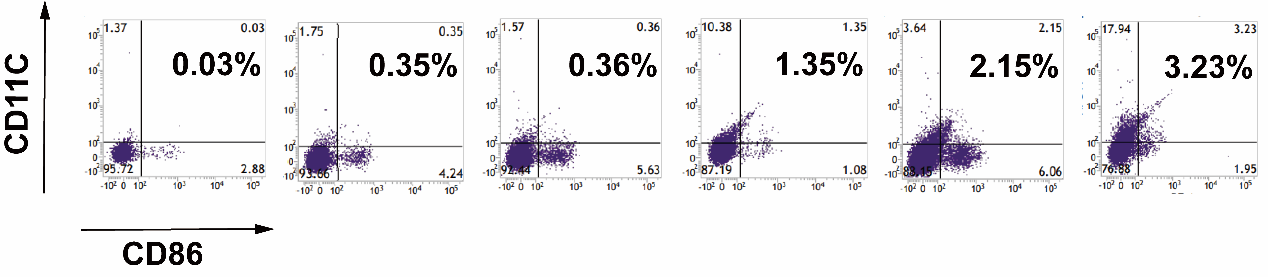
**

**Figure S20.** Flow cytometry analysis of CD86 (CD45+CD11b+CD11c+CD86+ as marker) in spleens after different treatments.


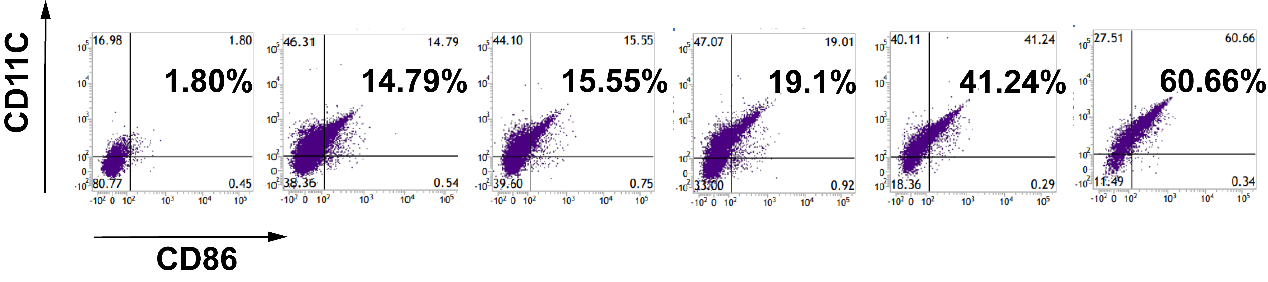


**Figure S21.** Flow cytometry analysis of CD86 (CD45+CD11b+CD11c+CD86+ as marker) in tumor tissues after different treatments.

**
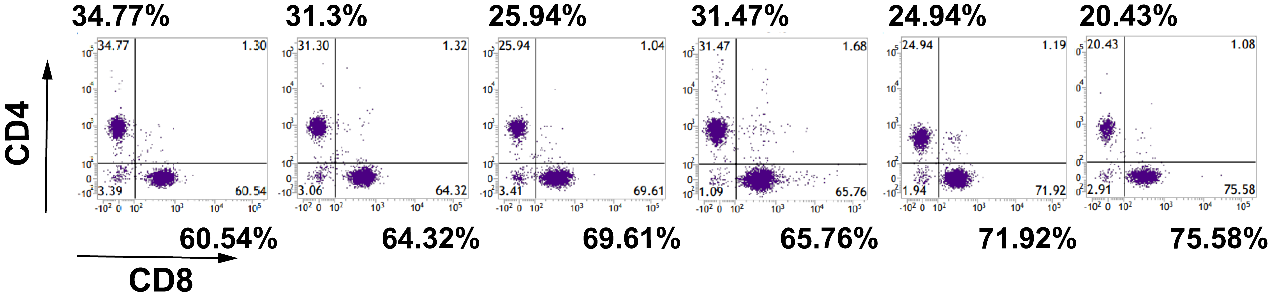
**

**Figure S22.** Flow cytometry analysis of CD4+ (CD3+CD4+ as marker) and CD8+ (CD3+CD8+ as marker) T cell populations in the spleens after different treatments.
